# Phase transitions as intermediate steps in the formation of molecularly engineered protein fibers

**DOI:** 10.1038/s42003-018-0090-y

**Published:** 2018-07-02

**Authors:** Pezhman Mohammadi, A. Sesilja Aranko, Laura Lemetti, Zoran Cenev, Quan Zhou, Salla Virtanen, Christopher P. Landowski, Merja Penttilä, Wolfgang J. Fischer, Wolfgang Wagermaier, Markus B. Linder

**Affiliations:** 1 Department of Bioproducts and Biosystems, School of Chemical Engineering, Aalto University, 02150 Espoo, Finland; 2 Department of Electrical Engineering and Automation, School of Electrical Engineering, Aalto University, 02150 Espoo, Finland; 3 VTT Technical Research Centre of Finland Ltd., 02150 Espoo, Finland; 4 Sappi Papier Holding GmbH, 8101 Gratkorn, Austria; 5 Department of Biomaterials, Max Planck Institute of Colloids and Interfaces, 14476 Potsdam, Germany

## Abstract

A central concept in molecular bioscience is how structure formation at different length scales is achieved. Here we use spider silk protein as a model to design new recombinant proteins that assemble into fibers. We made proteins with a three-block architecture with folded globular domains at each terminus of a truncated repetitive silk sequence. Aqueous solutions of these engineered proteins undergo liquid–liquid phase separation as an essential pre-assembly step before fibers can form by drawing in air. We show that two different forms of phase separation occur depending on solution conditions, but only one form leads to fiber assembly. Structural variants with one-block or two-block architectures do not lead to fibers. Fibers show strong adhesion to surfaces and self-fusing properties when placed into contact with each other. Our results show a link between protein architecture and phase separation behavior suggesting a general approach for understanding protein assembly from dilute solutions into functional structures.

## Abstract (web summary)

Pezhman Mohammadi et al. report the design of spidroin-inspired engineered proteins that separate from solution into coacervates with distinct properties depending on protein or salt concentration. These coacervates serve as intermediate assembly steps for self-fusing, adhesive fibers with useful biomechanical properties.

## Introduction

How biochemical structures are able to self-assemble in both a positional and temporal way is a key question for understanding the formation on functional entities such as cellular compartments, organelles, or in general biological materials. A general understanding is emerging that highlights phase transitions of biological macromolecules as a key mechanism for formation of such structures^1^.

The emerging understanding of phase separation in the formation of biological structures emphasizes the importance of mechanisms of colloidal interactions and phase separations to describe structural organization as a complement to our understanding of lock-and-key-type intermolecular interactions that involve more specific molecular recognition events^2^. Phase separation is a route toward the assembly of several biological structures, such as intracellular organelles, cellular compartments, fibrous structures, and organization in lipid membranes^1–4^. When a solution undergoes liquid–liquid phase separation, it separates into two immiscible liquid fractions that differ in composition. The phase separation occurs when conditions change so that macromolecule–macromolecule interactions becomes relatively stronger and overcome the entropic tendency to remain homogenously mixed^5^. The phase separation is dependent on conditions such as concentration, pH, ionic components, and other macromolecular solutes, and results in condensed biopolymer assemblies that are not stabilized by amphiphiles or membranes^6–8^.

The liquid–liquid phase separation of biological macromolecules is often referred to as coacervation^1,9–11^. The term complex coacervation refers to involvement of polyelectrolytes forming complexes together. Often such a complex coacervate is formed by the interaction of two different polyelectrolytes. In some cases, coacervates are formed by a single polyelectrolyte. These events have been referred to as self-^12,13^, elementary^14^, one-component^15^, or simple^16^ coacervation.

In addition to the role of phase separation for the formation of cellular structure, the process has been identified as a key step in the formation of many biological materials. The role of coacervates formed by liquid–liquid phase separation has been intensively studied, especially in the field of understanding the function of underwater adhesives by marine organisms^17,18^. In studies based on mussel and sandcastle worm adhesives, it was found that the coacervation step preassembles the protein constituents of the adhesives, which in combination with the low surface energy and cohesiveness results in a very efficient function^19–22^. The role of coacervates in the formation of biological materials such as squid beak has also been described, in which again the preassembled state of proteins and low surface energy of the coacervate leads to efficient infiltration of a scaffold and subsequently to the formation of mechanically excellent structures^12,13^. Also for tropoelastin assembly has it been shown that a coacervate step leads to structural assembly^4,23^. Common to all these material assembly processes seems to be that the high polyelectrolyte concentration within coacervates is associated with an advantageous pre-assembly due to a molecular structuring within the coacervate^14,24^.

There is also an increasing interest in applying biological components and methods to make materials and devices, giving for example biomaterials with useful mechanical properties, sensors, and even adhesives. Proteins have a substantial potential for such future sustainable and advanced functional materials. The wide possibility for precise design on multiple scales, together with the numerous examples in nature available to us as models give virtually endless possibilities for approaches and potential use. Protein-based materials are becoming increasingly feasible with the expanding knowledge of sequences, the ease of gene synthesis, cloning strategies, and biological production. However, the assembly of proteins differ markedly from those of synthetic polymers^25^ and one of the main obstacles on this path is that we still lack much in understanding of the processes by which materials are assembled and form their functional molecular interactions, both in temporal and structural hierarchy^12,21,26–30^.

In this work we have approached the general problem of how to direct the assembly of highly engineered proteins toward functional states from a biological materials perspective. Following the overall structural arrangement of spider silk proteins (i.e., spidroins)^31,32^, we produced engineered proteins that were combined from parts from unrelated sources and had an overall 3-block architecture, with a repetitive block in the middle, flanked by two relatively small folded domains at each terminus. The repetitive middle-block was based on the spidroins from *Araneus diadematus*^33^ and the terminal domains were chosen for having roughly the same size as native spidroin terminal domains^32^ and possible functional use in linking proteins with other material components or with other proteins. One type of terminal domain was a cellulose-binding module (CBM)^34^ and the other was the SpyCatcher^35^ which is able to form covalent linkages to specific peptide tags. We have explored how the overall design architecture of proteins affect liquid–liquid phase separation in self-coacervating systems, and importantly under which circumstances the formed coacervates function as an intermediate step toward functional assembly. The results show that phase separation is a key intermediate in the formation of fibrous structures by these silk-inspired proteins and that a careful balance of interactions between the macromolecules must be maintained during assembly.

## Results

### Protein architecture

**Fig. 1:**
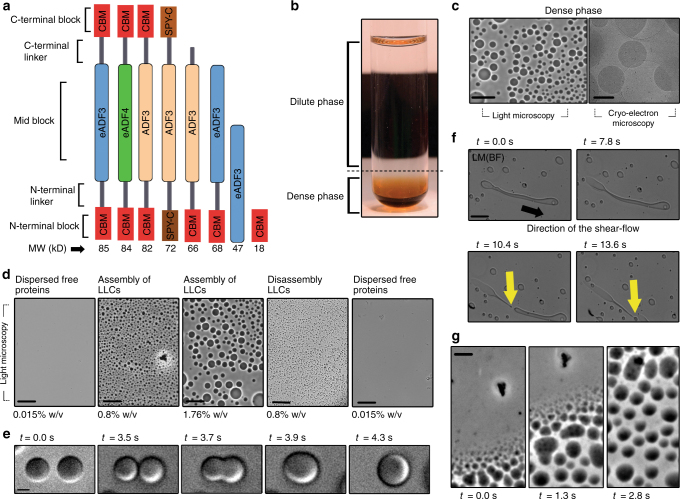
Structure and liquid–liquid phase separation of 3-block proteins. **a** Schematic representation of the proteins used in this study (MW, molecular weight). **b** Centrifugation of the liquid–liquid phase separated protein in pure water with a dilute clear protein solution on top and a dense translucent protein phase on the bottom. **c** Inverted light microscopy and cryo-transmission electron microscopy images of the dense phase showing spherical liquid-like coacervates (LLC) with diameters of 1–15 µm (scale bars are 20 µm for the light microscopy images and 1 µm for the cryo-EM image). **d** Light microscopy images showing the development of LLCs for CBM-ADF3-CBM. As the overall concentration of the solution increased to 0.8% w/v, small LLCs emerged in the dense phase and as the overall concentration of the solution increased further to 1.8% w/v LLCs grew larger in size. Dilution led to a dissociation of the LLCs (scale bars are 20 µm). **e** Bright-field light microscopy images showing time frames of the fusion of two individual droplets (scale bar is 1 µm). **f** Deformation of a single LLC droplet under shear flow and the formation of a fluid thread. The black arrow shows the direction of the flow. The yellow arrow shows the point at which the fluid thread breaks and a satellite droplet emerges (scale bar is 10 µm). **g** Time frames from inverted light microscopy showing adhesion and assembly of LLCs on the surface of a glass slide (scale bar is 2 µm)

Proteins were designed with a general 3-block architecture (Fig. 1a and Supplementary Fig. 1), having a repetitive mid-block and two terminal blocks, one at each end of the polymer. Variants of this block theme were made by either replacing blocks with alternative ones or by omitting individual blocks. Three different spidroin sequences were used as mid-blocks. First, a part of the ADF3 dragline silk sequence from *A. diadematus*^36^. Second, an engineered version of ADF3 called eADF3 consisting of a repeating consensus sequence^33^. Third, an engineered version of ADF4 dragline silk sequence from *A. diadematus*, called eADF4^37^. Two different types of terminal blocks were used, one was a thermally stable protein known as a CBM from the *Clostridium thermocellum* cellulosome^34^. Using this terminal block, proteins CBM-ADF3-CBM, CBM-eADF3-CBM, and CBM-eADF4-CBM were made. Another globular and stable, but non-homologous domain, SpyCatcher (SPY_C), that has been engineered from the fibronectin-binding protein FbaB of *Streptococcus pyogenes*^35^, was used as an alternative terminal block to form SPY_C-ADF3-SPY_C. In addition, variants were made with 2-block and 1-block architectures for eADF3, one with eADF3 attached to a single N-terminal CBM called CBM-eADF3, and the isolated eADF3 without added terminal-blocks. A variant CBM-ADF3, a single 1-block CBM without any repetitive block, and a single 1-block eADF3 were also produced. A summary of the constructs is provided in Supplementary Table 1 and the amino acid sequences can be found in Supplementary Data 1.

### Liquid–liquid phase separation

It was initially observed, that when concentrating solutions of the purified 3-block proteins CBM-ADF3-CBM and CBM-eADF3-CBM, a self-coacervation occurred. This liquid–liquid phase separation occurred when a solvent exchange into pure water (Milli-Q) had been made for the proteins (Fig. 1b). At a protein concentration of 0.015% w/v (1.7 µm), solutions remained clear. However, upon increasing the overall protein concentration to above 0.8% w/v (0.1 mM), solutions become slightly translucent, indicating the formation of condensed protein assemblies. We observed a phase separation into two distinct liquid phases by centrifugal separation, with a dilute clear solution on top and a dense phase on the bottom (Fig. 1b).

When the dense phase was analyzed by light and cryo-electron microscopy, it was observed that it contained viscous spherical and ellipsoid-shaped coacervates with a diameter of 1–15 µm, while the upper phase showed no detectable structures (Fig. 1c and Supplementary Movie 1). A liquid-like behavior of the coacervates was indicated by their dynamic nature. The term liquid-like coacervate (LLC) was used to describe the protein droplets formed in this way, in order to distinguish them from another form, described below. They formed and disassembled in a reversible concentration-dependent manner, with droplets growing by coalescence (Fig. 1d, e, and Supplementary Fig. 1). The LLC droplets were easily deformed by flow and the droplets could be broken up by mixing (Fig. 1f). In some cases, the coacervates were observed to adhere to the surface of glass slides (Fig. 1g).

The 3-block SPY_C-ADF3-SPY_C also formed indistinguishable LLCs in water, but only at a 2.5 times higher concentration (1.8% w/v, 0.25 mM) (Supplementary Fig. 1). Increasing the overall concentration further to 10% w/v and 30% w/v led to increasingly larger LLC droplets (Supplementary Fig. 2). The 2-block CBM-eADF3 and CBM-ADF3 also formed similar LLCs, but only at a still higher concentration of 8% (1.2 mM). The isolated eADF3 block remained soluble but no LLC formation could be observed even at 21.5% w/v (4.5 mM) concentration. The 1-block single CBM did not show liquid–liquid phase separation and started to precipitate at 2% w/v (1.7 mM) concentration (Supplementary Fig. 1). Studying the CBM-eADF4-CBM construct similarly was not possible as it aggregated easily (Supplementary Fig. 1).

### Formation of two types of coacervates

**Fig. 2:**
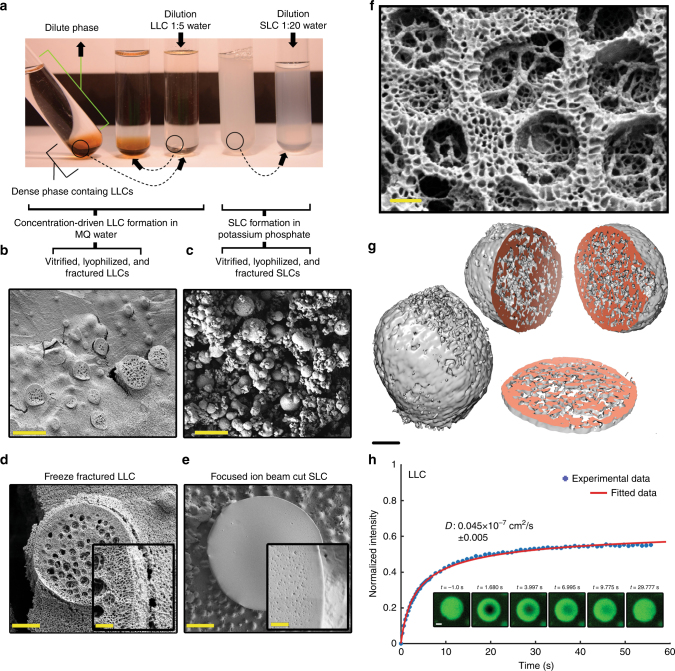
Phosphate-free and phosphate-containing conditions lead to different forms of coacervates. **a** Phosphate-free conditions lead to liquid-like coacervates (LLC) that show a reversible concentration-dependent formation (the three test tubes on the left). The tube most to the left is tilted to show that the dense phase is in a liquid form, while a potassium phosphate containing solution results in solid-like coacervates (SLC) that did not show dissociation during dilution (the two test tubes on the right). **b** Scanning electron micrograph of liquid ethane-propane vitrified and fractured specimens is LLC and **c** is the phosphate-induced SLC (scale bars 10 µm). In order to remove potassium phosphate, the SLC was washed three times with water before lyophilizing. **d**, **e** Images show differences in the internal structure of individual LLC and SLC droplets. The LLC was freeze-fractured while a focused ion beam was used to split the SLC (scale bars are 2 µm and 1 µm for the magnified inserts). **f** A high-magnification SEM image of the internal structure of a LLC droplet shows details of an internal bicontinuous network (scale bar 200 nm). **g** Electron tomography of SLC shows a porous bicontinuous structure (scale bar 30 nm). **h** FRAP experiments for a LLC coacervate. The inserts show droplets before and after bleaching at different time points (scale bars 2 µm). Additional data on SLC are shown in Supplementary Fig. 6

**Fig. 3:**
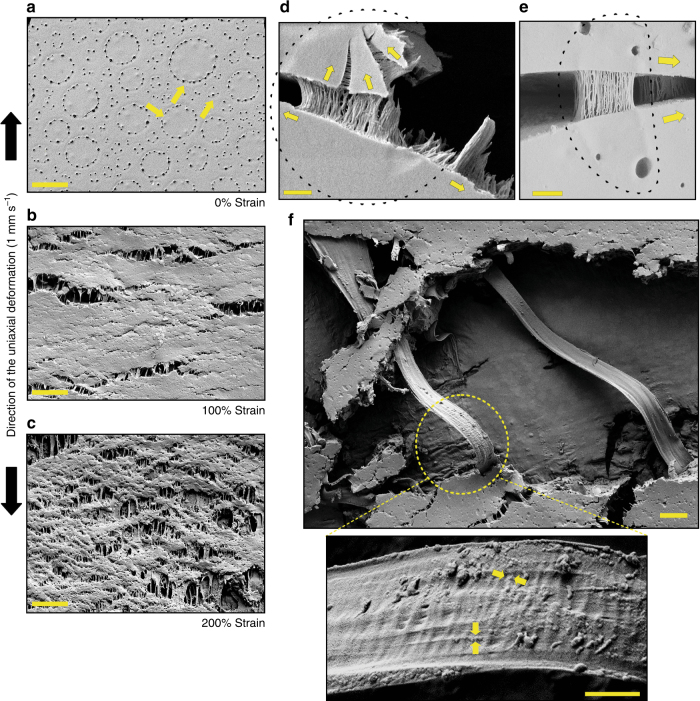
Cracks in semi-dry LLC droplets led to the formation of bridging filaments. **a** A thin film casted from a LLC containing solution with an overall protein concentration of 30% w/v (yellow arrows indicate individual LLC droplets in the unstrained film). Upon straining the semi-dried film to 100% (**b**) and 200% (**c**), long filaments appeared in cracks in the film in the direction of stretching (scale bar is 20 μm). **d**, **e** High-magnification SEM images from two individual micrometer sized LLCs (CBM-eADF3-CBM) embedded in a continuous matrix of non-coacervated protein. Upon pulling, bundles of nanometer size filaments bridging the cracks formed orthogonally to the direction of the crack (scale bar is 200 nm). As an aid to interpretation, the edges of the LLC droplet are marked with a dashed line and yellow arrows show the direction of crack propagation. **f** SEM micrograph of large LLCs with considerable plastic deformation pulled into a single and continuous filament (scale bar is 2 μm). At high magnification, nanometer size stripes became apparent both along and across the axis of the filaments (scale bar is 200 nm). Yellow arrows indicate striping patterns

We noted that self-coacervation occurred in a distinctly different way compared to LLCs if strong kosmotropes, such as K^+^ and PO_4_^−3^ were present. At high phosphate concentration, protein solutions became turbid (Fig. 2a). A phase diagram for the phosphate-dependent coacervation was measured (Supplementary Figs. 3, 4). This shows coacervation starting at about 200 mM phosphate, depending on protein concentration. At higher protein concentration, the coacervates required lower potassium phosphate concentrations to form. At 500 mM potassium phosphate this occurred for CBM-eADF3-CBM and CBM-ADF3-CBM already at concentrations of 0.025% w/v and 0.035% w/v, respectively, and for CBM-eADF3 and CBM-ADF3 at 0.03% w/v. Electron and light microscopy showed the presence of clusters of microspheres with a size range of 0.1–1 µm (Supplementary Figs. 3, 4). These phosphate-induced coacervates showed properties distinctly different from LLCs, and the term solid-like coacervate (SLC) was used to describe them. The SLCs were transparent in the light microscope (Supplementary Fig. 4). Compared to LLCs, the SLCs had more regular spherical shapes, they were typically smaller in size, they did not show coalescence nor deformability. Fluid threads did not form under flow and they did not stick to glass surfaces. Importantly, SLC formation was not reversible by dilution (Fig. 2a). Scanning electron microscopy was used to characterize differences between LLCs and SLCs further. LLCs could be freeze-fractured (Fig. 2b), while SLCs did not break up during freeze-fracturing, but instead the samples fractured at the outer surface of the SLC spheres (Fig. 2c). Their internal structure was imaged by splitting the droplets by a focused ion beam and by electron tomography. Both LLC and SLC showed porous bicontinuous networks but the SLC had a denser appearance (Figs. 2b, 3, Supplementary Fig. 5). We further quantified the physical properties of LLC and SLC by measuring the diffusivity of their constituent proteins by fluorescent recovery after photobleaching (FRAP). Lys residues in the CBMs allowed labeling with a fluorescent dye (Oregon Green 488). After coacervate formation, a small area (*r* = 2.5–5 µm) in droplets of LLC and SLC were bleached using a laser. Based on the rate of fluorescence recovery we could estimate diffusion coefficients (Fig. 2e). LLCs of CBM-eADF3-CBM had a coefficient (*D*) of 0.045 × 10^−7^ cm^2^ s^−1^ ± 0.005, while SLC formed by the same protein had a much lower *D* of 0.003 × 10^−7^ cm^2^ s^−1^ ± 0.001. Protein in the surrounding solution had a *D* of 1.18 × 10^−7^ cm^2^ s^−1^ ± 0.078. The diffusion of CBM-eADF3 in LLCs (*D* = 0.043 × 10^−7^ cm^2^ s^−1^ ± 0.017) compared to CBM-eADF3-CBM were within the confidence intervals of each other (Supplementary Fig. 6).

Additionally, we analyzed LLC and SLC coacervates using Fourier transform infrared spectroscopy (FTIR) by investigating signals in the range of 1000–1700 cm^−1^ (amide-I, II, and III bands) (Supplementary Fig. 6). The FTIR spectra of LLCs showed a major amide I band at 1630 cm^−1^ (associated with a bending C=O vibration), whereas SLCs showed a higher intensity major amide I band with the peak at 1632 cm^−1^ as well as a prominent amide II band at 1549 cm^−1^ (N–H). In addition, SLCs showed an amide III band at 1247 cm^-1^ (C–N) as a sharp and high intensity peak, whereas LLCs had a lower intensity peak at 1243 cm^−1^. FTIR spectra for LLC with having 0.5 M NaCl were identical to spectra of LLCs without NaCl (Supplementary Fig. 6).

### Bridging over cracks in semi-dry LLC droplets

LLC droplets were clearly identifiable by SEM in thin films (thickness of ~30–40 µm) that were made by casting a LLC solution with an overall concentration of 30% w/v on a solid support such as Parafilm and drying it. The texture of the films indicated LLC droplets with diameters of 5–20 µm dispersed throughout the film (Fig. 3a). Small perforations were clearly arranged around and defining the edges of the LLCs. We hypothesize that these perforations possibly form due to differences in the water evaporation rate between LLCs and the surrounding dispersed protein. When the film was stretched while still not completely dry, the formation of filament structures that were bridging the cracks could be observed (Fig. 3b, c). When using LLC samples of lower concentration (2% w/v) with droplets occurring more sparsely, we could observe how filament structures evolved when cracks passed through LLC droplets (Fig. 3d, e and Supplementary Fig. 7). The distinct bridging over the cracks and their orthogonal orientation in relation to the propagation of the cracks showed that filaments formed during cracking and were not present initially in the droplets. If individual droplets were allowed to grow large, filaments of lengths exceeding tens of micrometers were observed. Especially in these long filaments, a distinct striping pattern was observed both laterally and longitudinally with a periodicity of 20 nm (Fig. 3f). Polarized microscopy indicated second and/or third degree of molecular orientation (Supplementary Figs. 8, 9). The bridging over cracks was only observed for LLCs of CBM-ADF3-CBM, CBM-eADF3-CBM, and SPY_C-ADF3-SPY_C but not for CBM-eADF3, CBM-ADF3 nor eADF3, i.e., only for 3-block proteins, not 2-block or 1-block variants. We never observed bridging filaments for SLCs of any protein.

### Formation of single fibers

**Fig. 4:**
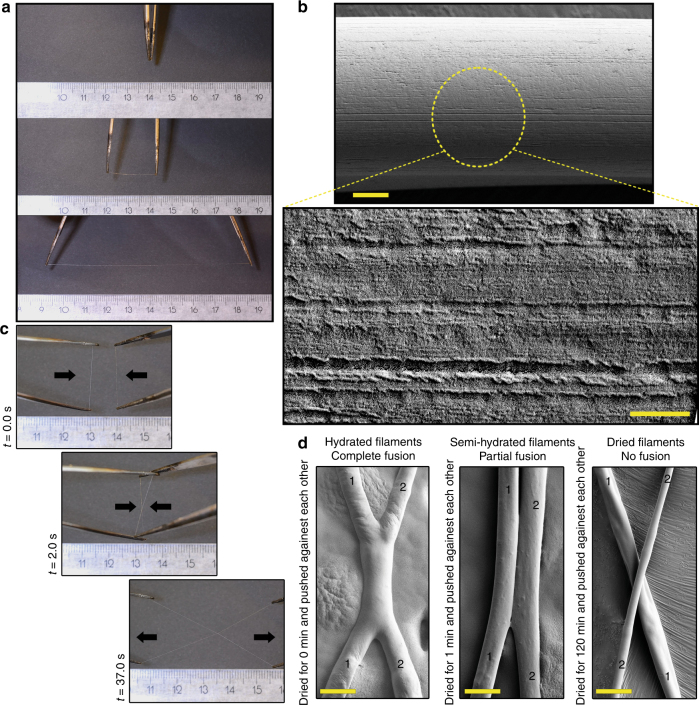
Dry spinning of 3-block architecture proteins into fibers at ambient temperature, pressure, and using water as the solvent. **a** Rapid formation of fibers by extending a 10–15 µL aliquot of LLC at 70–75% w/v. **b** SEM image of a single pulled fiber (scale bar 2 μm). High-magnification image from the surface of the fibers show a regular surface pattern with a periodicity of 20 nm (scale bar 200 nm). **c** Adherence of two freshly pulled fibers to each other. Junctions were strong enough to withstand stretching. **d** Electron micrographs showing the full fusion of two individual fibers placed in contact with each other. The fusing of fibers was dependent on water content, with dry fibers not fusing. The two original fibers are marked 1 and 2 (scale bar 20 μm)

Taking a 10–15 µL droplet of concentrated LLC (70–75% w/v) between the tips of a pair of tweezers and stretching it resulted in the formation of a single fiber (Fig. 4a, Supplementary Figs. 10, 11). The fibers easily extended to lengths of 5–10 cm, depending on the volume of protein. The diameter of the fibers ranged from 8 to over 200 μm depending on how much they were extended (Supplementary Fig. 11). When freshly drawn, the fibers showed a high elasticity, and when tension was released they retracted and could be pulled out again in multiple cycles (Supplementary Movie 2). Allowing the fibers to dry totally, we noticed that they became less elastic and more brittle. High-magnification SEM images revealed a surface pattern of lines with a regular spacing of 20 nm (Fig. 4b, Supplementary Figs. 11, 12). Polarized optical microscopy (Supplementary Fig. 13) showed the strongest partial orientation at the edges of the fibers indicating that crystallization was mainly located at the outer most layer of the fibers. Sometimes bead-like structures formed on the fibers during pulling (Supplementary Fig. 14). High-magnification phase-contrast images of these beads showed bundles of aligned filamentous structures extending through them.

As with the crack-bridging filaments, we could produce single fibers only for LLCs of SPY_C-ADF3-SPY_C, CBM-ADF3-CBM, and CBM-eADF3-CBM, but not for CBM-eADF3, CBM-ADF3, and never with SLCs of any proteins.

### Self-fusing and adhesive properties of fibers

**Fig. 5:**
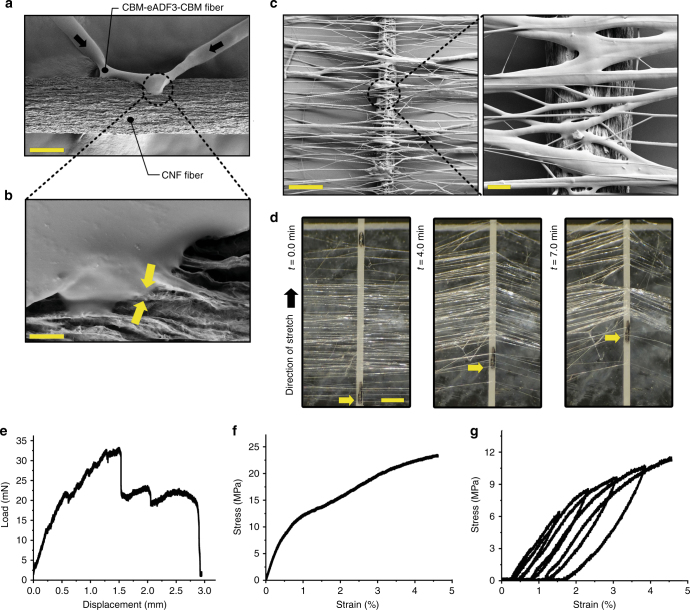
Adhesive properties of LLC fibers and mechanical testing of single fibers. **a** The attachment of a freshly drawn CBM-eADF3-CBM fiber to a cellulose fiber (CNF) (scale bar 50 μm) showing **b** how the fluid nature of the fiber leads to a melting in of the structures (scale bar 200 nm). Yellow arrows indicate the interface. **c** Stapling of a cellulose fiber using multiple LLC fibers. The insert shows how LLC fibers fuse together (scale bar 100 μm and 20 μm). **d** The stapling led to the attachment of the cellulose fiber to a PMMA surface that resisted pulling (scale bar 200 μm). The yellow arrow marks a spot on the fiber for the determining the displacement. **e** The in-plane adhesion force measured in a tensiometer, showed rupture of individual fibers before the final break-off. **f** Tensile testing of individual fibers showed a two-stage mechanism with an initial elastic deformation that was followed by a plastic deformation until final rupture. **g** Cyclic measurements were used to confirm the elastic/plastic regions of the tensiogram, with a yield point at 1% strain

When two freshly pulled fibers were placed in contact with each other, they fused together within a few seconds (Fig. 4c, Supplementary Fig. 15, and Movie 3). During drying, the ability of fibers to fuse successively decreased, and dry fibers did not fuse (Fig. 4d). This self-fusing property was also manifested as an adhesive behavior. Placing a freshly drawn LLC fiber on another fiber, such as cellulose, the LLC fiber partially fused into the rough cellulose fiber surface (Fig. 5a). The junction became highly adhesive (Fig. 5b). To further understand the adhesiveness of LLC fibers, several fibers were placed over a cellulose fiber, as if staple pinning the cellulose fiber with LLC fibers. As shown in Fig. 5c, the LLC fibers partially fused on the cellulose fiber and on the underlying poly-methyl methacrylate (PMMA) support. Placing this setup in a tensile tester, the in-plane pull-off force could be measured (Fig. 5d, e, Supplementary Fig. 16, and Movie 4). The fastened LLC fibers showed sequential rupture indicated by bumps in the force-distance curve until a final rupture. The pull-off force was 27–35 mN for a fiber fixed with 70 LLC fibers.

### The mechanical properties of individual fibers

The mechanical properties of dried LLC fibers at 50% relative humidity were measured in monotonic and step cyclic tensile tests (Fig. 5f, g and Supplementary Figs. 17–19). The general shape of the stress–strain curves for all constructs showed two different regions, first a linear elastic deformation regime which then was followed by a yield point and a region of plastic deformation until the fiber underwent catastrophic failure. The yield point was at about 1% strain. We compared mechanical properties by stress–strain curves of CBM-ADF3-CBM, CBM-eADF3-CBM, and SPY_C-ADF3-SPY_C but did not notice substantial difference between them. Fibers made from all three proteins showed mean values of 16 MPa for ultimate strength, 0.8 GPa for Young’s modulus, 4% for ultimate strain, and 0.7 MJm^−3^ for toughness (Supplementary Fig. 17). Cyclic measurements followed the trace of single loading curves and showed elastic recovery in each cycle so that the fiber recovered back to the starting point of the cycle. Further strain led to plastic deformation, until the fiber underwent catastrophic failure after 4–6 cycles (Fig. 5g and Supplementary Fig. 18).

The effect of the relative humidity was studied additionally at 25 and 80% relative humidity (Supplementary Fig. 19). Fibers were equilibrated at the relative humidity for a minimum of 2 h prior to measurement. At 25% relative humidity the fibers became stiffer and more brittle with 11 MPa strength, 0.8% strain, 1.3 GPa Young’s modulus, and 0.08 MJm^−3^ toughness. At 80% relative humidity the deformation of the fibers increased to 6.5% strain but they showed otherwise decreased properties, with 4.6 MPa ultimate strength, 0.13 GPa Young’s modulus, and 0.22 MJm^-3^ toughness. If the fibers were completely submerged in water they showed swelling and broke easily into multiple segments.

Measuring fiber properties directly after formation with water evaporation still ongoing showed highly ductile and deformable properties (Supplementary Fig. 20). The semi-dry fibers of CBM-ADF3-CBM showed 78 kPa in strength, 160 MPa for the Young’s modulus, 230% strain, and 74 Jm^−3^ in toughness. CBM-eADF3-CBM and SPY_C-ADF3-SPY_C showed corresponding values of 55 and 48 kPa strength, 144 and 153 MPa Young’s modulus, 198 and 210% strain, and 56 and 61 Jm^−3^ in toughness, respectively. A summary of all the mechanical properties can be found in Supplementary Tables 2–4.

### Molecular arrangements by WAXS

**Fig. 6:**
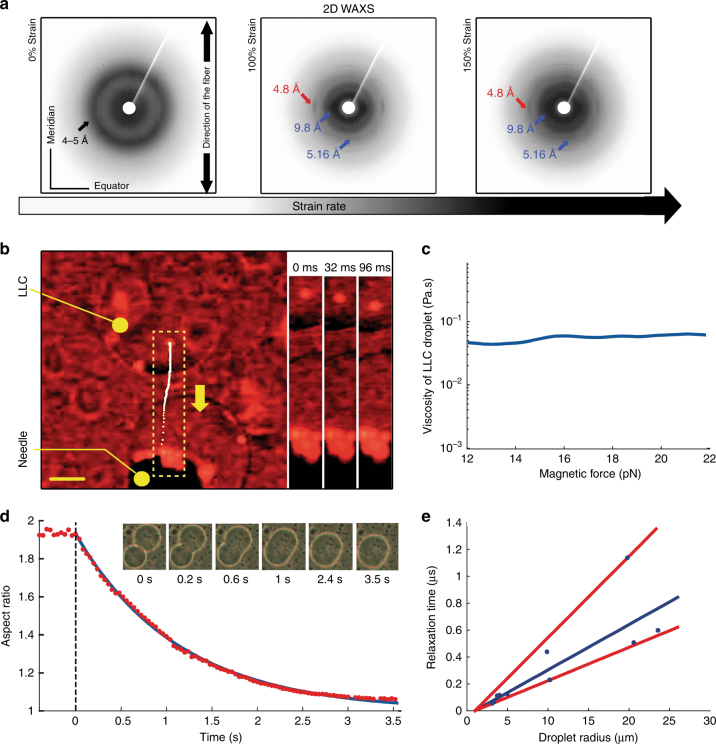
2D wide angle X-ray diffraction of the air pulled fibers and viscosity–surface tension estimation of LLC droplets. **a** 2D WAXS patterns for a bundle of LLC pulled fibers at 0, 100, and 150% strains. **b** Magnetophoretic movement of an encapsulated superparamagnetic (Fe_3_O_4_@PS) particle in LLC of CBM-eADF3-CBM (scale bar 50 μm). Box with the dashed line indicates the region of interest. The white dots denote the particle trajectory. The yellow arrow denotes the direction of the movement. **c** Viscosity as a function of the magnetic force. **d** Merging of two protein droplets and exponential decay fit of a merging event. **e** Relaxation time vs. final droplet radius for merging of LLCs of CBM-eADF3-CBM. The blue line is a linear fit of the data and the red lines represent boundaries of the inverse capillary velocity, *η*/*γ* ≈0.033 s μm^−1^

Synchrotron WAXS diffraction was used to analyze the ultrastructure of single fibers. Fibers were made by applying a drop of LLC between the tips of a pair of tweezers and placing the resulting fiber between the sample holders of a tensile testing device. In situ WAXS measurements were made on freshly prepared fibers strained to 0, 100, and 150% and on fibers that had been dried (Fig. 6a, Supplementary Figs. 21, 22). Freshly prepared fibers at 0% strain gave an azimuthal Debye ring as a halo at 4–5 Å corresponding to a characteristic scattering pattern of non-oriented polypeptide chains^38^. Stretching to 100% strain resulted in an equatorial peak at 4.8 Å indicating the presence of oriented β-sheets in the fiber direction^38–42^. A broad meridian arc at 5.16 Å corresponds to a partially ordered α-helical pitch. At the same strain, a broad peak with the highest intensity at 9.8 Å was also visible. This distance corresponds to the mean distance between α-helices, being consistent with a packing of helices against each other. Further stretching of the fiber to 150% resulted in the intensification of the peaks. Combined molecular modeling, steered molecular dynamic simulation (SMD), and scattering simulations were used to aid interpretation of the WAXS data (Supplementary Figs. 23–26).

The orientation and size of β-sheet crystallites were quantitatively determined from azimuthal broadening (FWHM) of the 4.8 Å equatorial diffraction using the Hermans orientation function^39,43^ and the Scherrer equation^41,44^. The value for the Hermans orientation parameter was maximally 0.76 for both CBM-eADF3-CBM and CBM-ADF3-CBM at 100% and increased to 0.82 and 0.81 at 150% strain indicating an increase of alignment along the tensile axis. The average crystallite size was 27.2–29.4 Å at 100% strain, and 29.1 and 29.4 Å at 150% strain for CBM-ADF3-CBM and CBM-eADF3-CBM, respectively (Supplementary Fig. 27).

### Viscosity and surface energy

Using a micro-rheology technique, we measured the viscosity of individual LLC droplets (Fig. 6b, c, Supplementary Fig. 28, and Movie 5). Fe_3_O_4_ in polystyrene (Fe_3_O_4_@PS) superparamagnetic microparticles with a diameter of 4.5 µm were first entrapped in LLC droplets. The movement velocity and path of the particles inside the LLCs were then tracked upon applying a magnetic field gradient from an electromagnetic needle positioned in close proximity. We found the viscosity of the LLCs to be around 50 mPa s. We then studied the dynamics of how two LLC droplets (with radii from 3 to 20 µm) coalesced completely and relaxed into a single sphere (Fig. 6d, e). The inverse-capillary-velocity which is the ratio between viscosity and surface energy was *≈* 0.033 s μm^−1^. This allowed us to calculate a value of 1.5 µN m^−1^ ± 0.5 for the interfacial energy for the LLCs.

## Discussion

Our results establish a clear role of liquid–liquid phase separation as an intermediate state in the formation of fibers by structurally engineered proteins, with the bridge-forming filaments that spanned cracks as a particularly clear manifestation. The formation of two different self-coacervated states, denoted as LLC and SLC, showed that the balance between surrounding conditions and protein architecture lead to different types of energy minima depending on solution conditions. It is an exceptional finding in this work that the same protein showed two different forms of coacervates. FTIR and FRAP show that the mechanisms for formation were different, but both forms still showed characteristic features of coacervates such as the sponge-like bicontinuous internal structure^14,24^. The diffusion rate of proteins within the LLCs was an order of magnitude higher than in SLCs, indicating a looser association between molecules within LLCs. Only LLCs could function as intermediates for fibers, showing that for subsequent structure formation the molecular interactions must be balanced in a metastable way. The stronger attractive forces induced by potassium phosphate in the SLC did not allow for subsequent assembly into fibers. The self-fusing property of fibers from LLCs also indicates that the proteins are in dynamic interaction with each other, i.e., the protein chains have a low barrier toward associating dynamically, possibly in an analogous way to in which interactions in self-healing materials are dynamic^45^. The LLC droplets had a very low surface energy of 1–2 μN m^−1^ and a relatively low viscosity of 50 mPa s, both of which are in the low end of what is expected for coacervates^7,46^.

The finding that modifying the overall protein architecture leads to different routes for subsequent phase behavior is not unexpected, but we are still in the initial steps of understanding the rules for such architectural design. There is a similarity between our 3-block architecture and the structure of spidroin proteins, in that spidroins have folded terminal blocks in their sequences^32,47^. However, details differ appreciably. The spidroin terminal domains contain switchers that trigger strong dimerization, and also the length of the repetitive middle part is noticeably longer in spidroins. CBMs were chosen as the subject to study as they are interesting as components in structural proteins as parts of molecularly designed cellulose-based materials^48^. However, as the unrelated protein SPY_C functioned similarly in liquid–liquid phase separation, we could demonstrate that these rules of design can have a more general validity and are not only a fortuitous function of the CBMs. The terminal domains can act to promote association between proteins for example by weak dimerization, or by enhancing entanglement of proteins by preventing the sliding of protein chains past each other. Such restriction of diffusion in concentrated solutions could enhance entanglement and thus promote phase separation.

Despite the role of the overall 3-block architecture, much of the intermolecular interactions seem to relate to the repetitive Ala-rich regions in the mid-block. Ala repeats can cause chain–chain interactions by interacting α-helix segments, or by β-sheet formation between strands. FTIR indicates a high content of α-helices in the LLC, which is consistent with the high propensity of Ala-stretches to form α-helices^49^. The high intensity of the amide I band in FTIR for SLCs compared to LLCs showed that the transition from LLC to SLCs is accompanied by the formation of β-sheet structures^50^. It is interesting that having 0.5 M NaCl with LLCs did not induce the formation of SLCs, as was the case for phosphate. The formation of β-sheet structures therefore seem particularly sensitive to the presence of phosphate, as has been suggested earlier^51^. It has also been suggested for native spidroins, that the assembly process is dependent on the nature of ions present^52^. The formation β-sheets was accompanied by a lower internal molecular diffusion rate as seen by FRAP, as would be expected because β-sheets are more efficient in interlocking the protein backbone chains. When LLCs were used to make air-pulled fibers, these were shown by synchrotron WAXS to contain components of clustered α-helices and β-sheet crystallites with a directionality following the orientation of the fibers. A transition of α-helices to β- sheets during the fiber extension is therefore indicated. Molecular orientation and induced crystallization was only observed for fibers stretched to 100 and 150% strain and not for fibers at 0% strain. The development of these during extension suggests a mechanically induced transition. Similar strain-induced α-helix bundling and β-sheet crystallization and molecular alignment responses have been observed in elastic deformation of several protein polymers containing stretches of Ala-residues^42,53–55^. This is also in line with earlier work on Ala-rich proteins demonstrating the substantial importance of mechanical stretching and elongational shearing and how this is sufficient to induce conformational transition and crystallization in vivo leading to domains containing β-sheets^36,56–58^. The combined observations from polarized microscopy, high-resolution scanning electron microscopy, and synchrotron WAXS also lead us to conclude that after fiber pulling there is a formation of a skin-core structure resulting from induced crystallization mainly at the outer most layer of the fiber, penetrating about 0.5–1 µm into the material. The fibers showed stress–strain curves with an initial elastic region followed by a region of plastic deformation in monotonic tensile measurement tests. Step cyclic measurements also showed the same elastic recovery as in the single loading curve. Both are indicative of toughening mechanisms that are also seen in other fibers, both natural ones and based on recombinant proteins^59,60^.

As the WAXS data indicate the presence of bundled α-helices in the freshly drawn fibers of LLC and FTIR suggested a higher β-sheet content in SLC vs. LLC, we find it feasible that bundling of α-helices is an important cohesive force within LLCs and the shifting of these structures toward β-sheet structures is responsible for the more arrested state in SLCs. This more arrested state would not allow drawing of fibers. This implies that a higher β-sheet content may be beneficial for final fiber properties, but not for initial fiber assembly.

The present work highlights the role of phase separations for the assembly of proteins into fibrous structures. As the process was conducted in vitro, conditions could be carefully controlled and led to the identification of functionally different self-coacervated assemblies depending on solution conditions. The use of molecularly engineered proteins allows understanding the link between protein architecture and assembly, and in future developments, the functions and properties of the terminal domains could be modified for mechanisms that are more robust for control of in vitro assembly processes. A full understanding of the protein structural features leading to phase separation into coacervates is also likely to bring new routes and understanding to the overall question of functional assembly in both cellular mechanisms, and for protein-based biological materials in general.

## Methods

### Cloning, expression, and purification in *Escherichia coli*

The DNA sequences encoding bacterial type three CBM from *Ruminiclostridium thermocellum* (Uniprot ID: Q06851)^34^, 12 repeats of residues 325–368 from the sequenced fragment of *Araneus diadematus* major ampulla gland silk fibroin 3 (Uniprot ID: Q16987) called eADF3^33,36,61^ (engineered sequence), and DNA sequence encoding residues 9–507 of ADF3 called ADF3 (wild-type sequence) were codon optimized and synthesized by GeneArt gene synthesis (Thermo Fisher Scientific) for expression in *Escherichia coli*. Three constructs were made using seamless golden gate cloning assembly of synthetic fragments on pEt-28a (+) (kanR) protein expression vector (Novagen) in frame with C-terminal 6×His-tag coding sequence for facilitating the purification^62–64^ and named CBM-eADF3-CBM, CBM-eADF3, and CBM. DNA sequence encoding ADF3 in frame with N-terminal and C-terminal CBMs was codon optimized for expression in *E. coli*, ordered as an intact piece from GeneArt gene synthesis (Thermo Fisher Scientific) and also inserted into the pEt-28a (+) (kanR) expression vector to make CBM-ADF3-CBM. DNA sequence encoding for engineered SpyCatcher^35^ (an E48K variant of SpyCatcher, from now on referred to as SpyCatcher or SPY_C) was codon optimized for expression in *E. coli* and ordered from GeneArt gene synthesis (Thermo Fisher Scientific). SPY_C-ADF3-SPY_C was constructed by replacing the CBMs at both the N-termini and C-termini of CBM-ADF3-CBM with SpyCatcher coding sequences by taking advantage of restriction enzyme sites designed in the original CBM-ADF3-CBM construct. To constructs eADF3 alone, codon optimized synthetic genes for expression in *Pichia pastoris* were ordered from Geneart. DNA sequence encoding engineered recombinant spider silk protein based on the sequence of ADF4 from *Araneus diadematus* fibroin (eADF4)^33,36,61^ in frame with N-terminal and C-terminal CBMs was also codon optimized for expression in *Pichia pastoris*, ordered as an intact piece from GeneArt gene synthesis (Thermo Fisher Scientific) and named CBM-eADF4-CBM. Both eADF3 and CBM-eADF4-CBM were then inserted into the pPICZ-α (Invitrogen) expression vectors. Further detail on cloning, expression, and purification can be found in the Supplementary Methods.

### LLC assembly and SLC formation

LLC assembly was initiated by controlled dehydration and gradual concentration of freshly purified dilute protein samples. During concentration, there was a protein-rich LLC phase formed which was collected from the solution for further characterization. Unless otherwise stated proteins were in Milli-Q water. Centrifugal concentrators (Vivaspin20, Sigma-Aldrich) at 845 r.c.f were used for concentration. Sample preparation and imaging were performed at 20–25 °C. SLC formation carried out by mixing the fusion proteins with potassium phosphate (pH 7.4) at final w/v concentration of 0.05% to final molar concentration of 500 mM. To study the phase diagram different concentrations of CBM-eADF3-CBM constructs and potassium phosphate (pH 7.4) were tested.

### Microscopy of the materials

The following microscopy setups were used: (1) Axio observer inverted microscope (Carl Zeiss, Germany) equipped with motorized stage, AxioCam MRm camera (Zeiss), a ×100/numerical aperture, and Zeiss AxioVision software. Images were further processed with ImageJ^65^ or ImageJ Fiji (version 1.47d)^66^. (2) Polarized microscopy imaging was done using a Leica DM4500 P LED polarized optical microscope for the qualitative observation and to study birefringence. (3) Scanning electron microscopy was performed using either a Zeiss FE-SEM or a HR-TEM (JEOL JEM-2800) field-emission microscope with variable pressure, operating at 1–1.5 kV. (4) High-resolution transmission cryo-electron microscopy imaging was carried out using JEM-3200Fsc field-emission microscope (JEOL) operated at 300 kV in bright-field mode with Omega-Zero-loss energy filter with a 20 eV slit. (5) A Veeco dimension 5000 AFM instrument was used and images were recorded in tapping mode in air with scan rates of 0.8–1 Hz with a FASTSCAN-B cantilever. (6) Dual beam focused ion beam/SEM (FEI HELIOS) apparatus was used for sectioning and imaging fibers and also beads on them. Further details are found in the Supplementary Methods.

### Attenuated total reflectance FTIR

A Unicam Mattson 3000 FTIR spectrometer equipped with PIKE Technologies GladiATR (with diamond crystal plate) was used for recording FTIR. All spectra were scanned within the range of 400–4000 cm^−1^, with a total of 32 scans and a resolution of 32 cm^−1^.

### Fluorescence recovery after photobleaching

Surface exposed Lys residues of the terminal CBM domains in CBM-eADF3-CBM and CBM-eADF3 were selectively labeled with Oregon Green 488 (carboxylic acid, succinimidyl ester, 6-isomer; Thermo Fisher). FRAP experiments were carried out using a Leica TCS SP5 upright confocal microscope with FRAP booster (Leica DM5000) equipped with argon (Blue: 488 nm) laser and DD488/561 dichroid beam splitter at 63×/1.2 water objective. Spots with diameters of 2.5–5 µm were excited with the laser. Emission was passed through 88/561 dichroid and detected with standard photomultiplier tube. Data were analyzed with Leica AF Lite–TCS MP5 software and processed further using Matlab. Fitting of the data was carried out according to Eq. (1)^67^. Details can be found in the Supplementary Methods.

1 $$D = \left( {V_0^2\gamma _D{\mathrm{/}}4\gamma _F^2} \right)\left[ {\left( {\tau _{1/2}^C} \right)^2{\mathrm{/}}\tau _{1/2}} \right].$$

### Synchrotron wide-angle X-ray scattering and in situ stretch hold deformation

In situ stretch-hold deformation and wide-angle X-ray diffraction experiments were carried out at the µSpot beamline at BESSY II synchrotron source (Berliner Elektronenspeicherring-Gesellschaft für Synchrotronstrahlung, Helmholtz-Zentrum Berlin, Germany) equipped with a custom-made tensile tester. Intensities around the equator and meridian were integrated radially using combination of DPDAK (with built-in algorithm from Fit2D and pyDAI software package) and jSAX programs after subtraction of air scattering and dark current from the diffractogram. Mean crystallite size values were calculated from azimuthal broadening (full width half maximum, FWHM) of the 4.8 Å equatorial peak fitted by Gaussian function according to Scherrer’s equation (Eq. (2)) including the instrumental broadening correction^41,44,68,69^, where $\beta$ is the FWHM. The axial orientation of the crystallite was also calculated from azimuthal broadening of the 4.8 Å peak using Herman’s orientation parameter according to Eqs. (3) and (4)^70,71^. Subsequently, thin azimuthal intensity profiles at 4.8 Å equatorial reflection were extracted by sector-wise integration after masking the diffractogram to show only the corresponding reflection ring and fitting the data with Gaussian curve. The orientation of the nanocrystals was then quantified by extracting the FWHM ($\Phi$). Herman’s orientation parameter is 0 for no preferred orientation in the filaments and 1 if all crystals are aligned perfectly with respect to each other in direction of filament axis^38^. Matlab and OriginLab were used for processing and presenting the data. Details are found in the Supplementary Methods.

2 $$L = \frac{{0.9{\mathrm{\lambda }}}}{{\beta \cos \theta }}$$

3 $$S = \frac{3}{2}{\mathrm {cos}}^2{\it{\Phi }} - \frac{1}{2}$$

4 $${\mathrm {cos}}^2{\it{\Phi }} = \frac{{\mathop {\sum}\nolimits_0^\pi I \left( {\it{\Phi }} \right){\mathrm {sin}}{\it{\Phi }}{\mathrm {cos}}^2{\it{\Phi }}}}{{\Sigma ^\pi I\left( {\it{\Phi }} \right){\mathrm {sin}}{\it{\Phi }}}}.$$

### Tensile testing of protein filaments

Single filaments, post-stretched at 150%, were placed across a laser-cut acrylic glass with a gauge length of 5 mM (Supplementary Fig.  1). Filaments where then fixed tightly to the acrylic glass by placing a small droplet of fast-cold-curing Loctite^®^ adhesive to prevent any slippage error during the measurement. All the specimens were tested using a specialized custom-made micromechanical testing device^72,73^. Defective fibers were eliminated before mechanical measurement. SEM was used to accurately measure the cross section of each filament. Upon measurement, sample holders with already fixed filaments were mounted to the micro bond tester and the bridges between the moving part (A) and fix part (B) of the sample holder were melted using a hot wire to free the fiber for the mechanical measurement at deformation rate of 2 µm/s until fiber rupture. All the measurements were carried out at 50% relative humidity and 23 °C. To study the effect of humidity, a set of filaments was measured at 25 and 80% relative humidity. All the filaments were stabilized for minimum of 2 h at each relative humidity. Freshly prepared semi-dried (dried for only 5 min) filaments were also measured at 50% relative humidity using the same sample holders and experimental setup except with deformation rate of 5 µm/s. Matlab^®^ (R2016) and OriginLab^®^ (2016) were used for processing the data.

### Data availability

Primary data are available^74^ for download at https://zenodo.org/record/1202316#.Ww-5Joq-n9Q. This upload contains raw and unprocessed data files, including tensile test, diffraction, simulations, surface tension measurement, viscosity measurements, amino acid sequences, and movies.

## Electronic supplementary material

Supplementary Information

Description of Additional Supplementary Files

Supplementary Movie 1

Supplementary Movie 2

Supplementary Movie 3

Supplementary Movie 4

Supplementary Movie 5

Supplementary Data 1
