# Supplementary material for: Phase transitions as intermediate steps in the formation of molecularly engineered protein fibers
Source: Commun Biol. 2018 Jul 2;1:86. doi: 10.1038/s42003-018-0090-y (PMC6123624; doi:10.1038/s42003-018-0090-y)
Supplement: Supplementary file 1 — Supplementary Information [file 42003_2018_90_MOESM1_ESM.pdf]

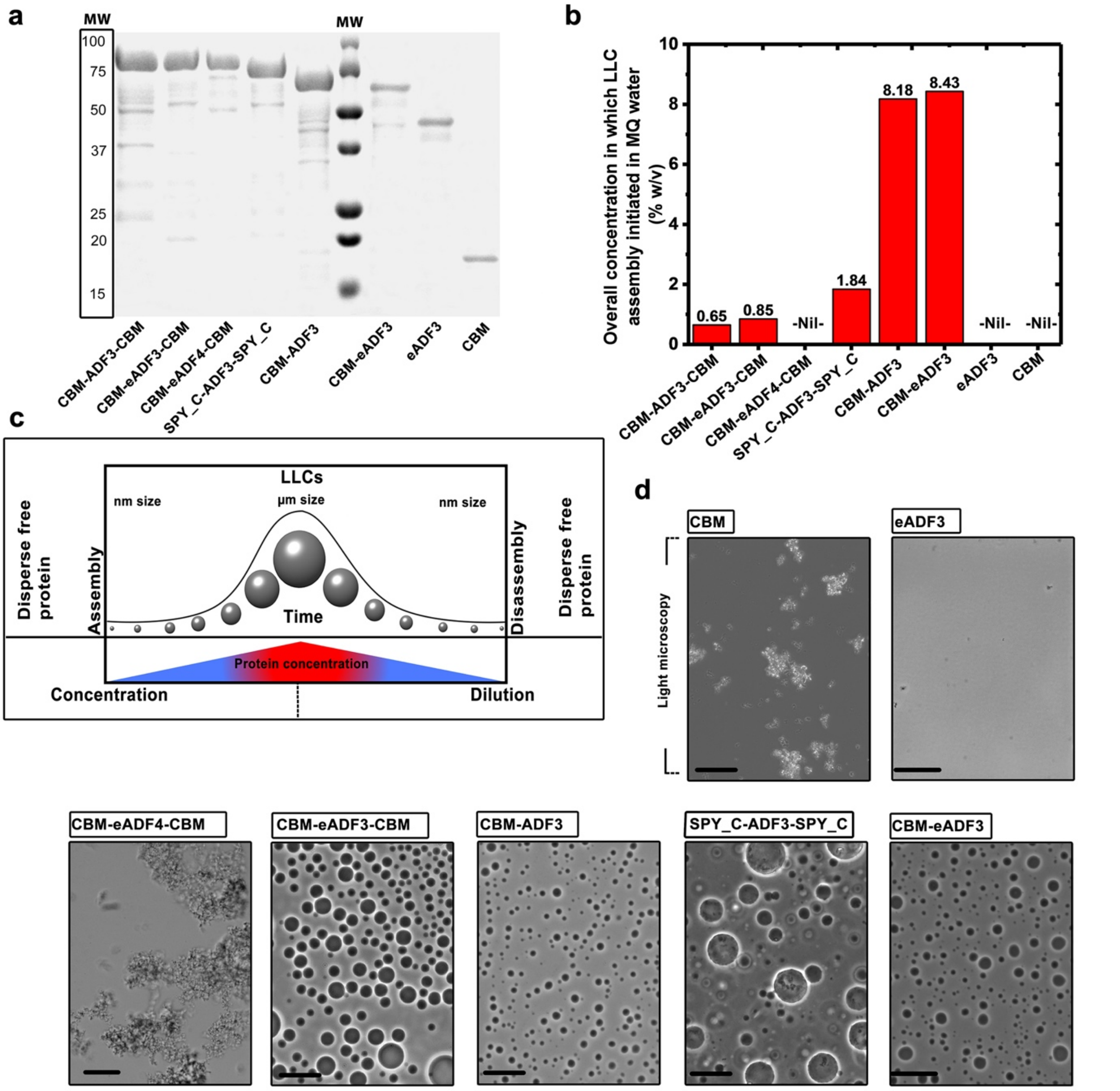


**Supplementary Figure 1.** LLC formation by the 3 block architecture proteins. (a) Sodium dodecyl sulfate-polyacrylamide gel electrophoresis (SDS-PAGE) separation of all the constructs (MW: molecular weight in kD). (b) Concentration threshold in which LLC assembly initiated for different constructs. (c) Schematic illustration of concentration dependency of LLC formation. (d) Phase contrast light microscopy images of concentrated solutions of different constructs. CBM-eADF3-CBM, CBM-ADF3-CBM, SPY_C-ADF3-SPY_C, CBM-ADF3, and CBM-eADF3 undergo LLCs formation at concentrations ranging from 0.1 to 1.4 mM. Studying the CBM and CBM-eADF4-CBM was not possible

as both constructs were highly prone to aggregate during the processing. In the other hand eADF3 remained entirely soluble with neither LLC assembly nor aggregation observed at 1.7 mM concentrations (scale bar is 20 µm).

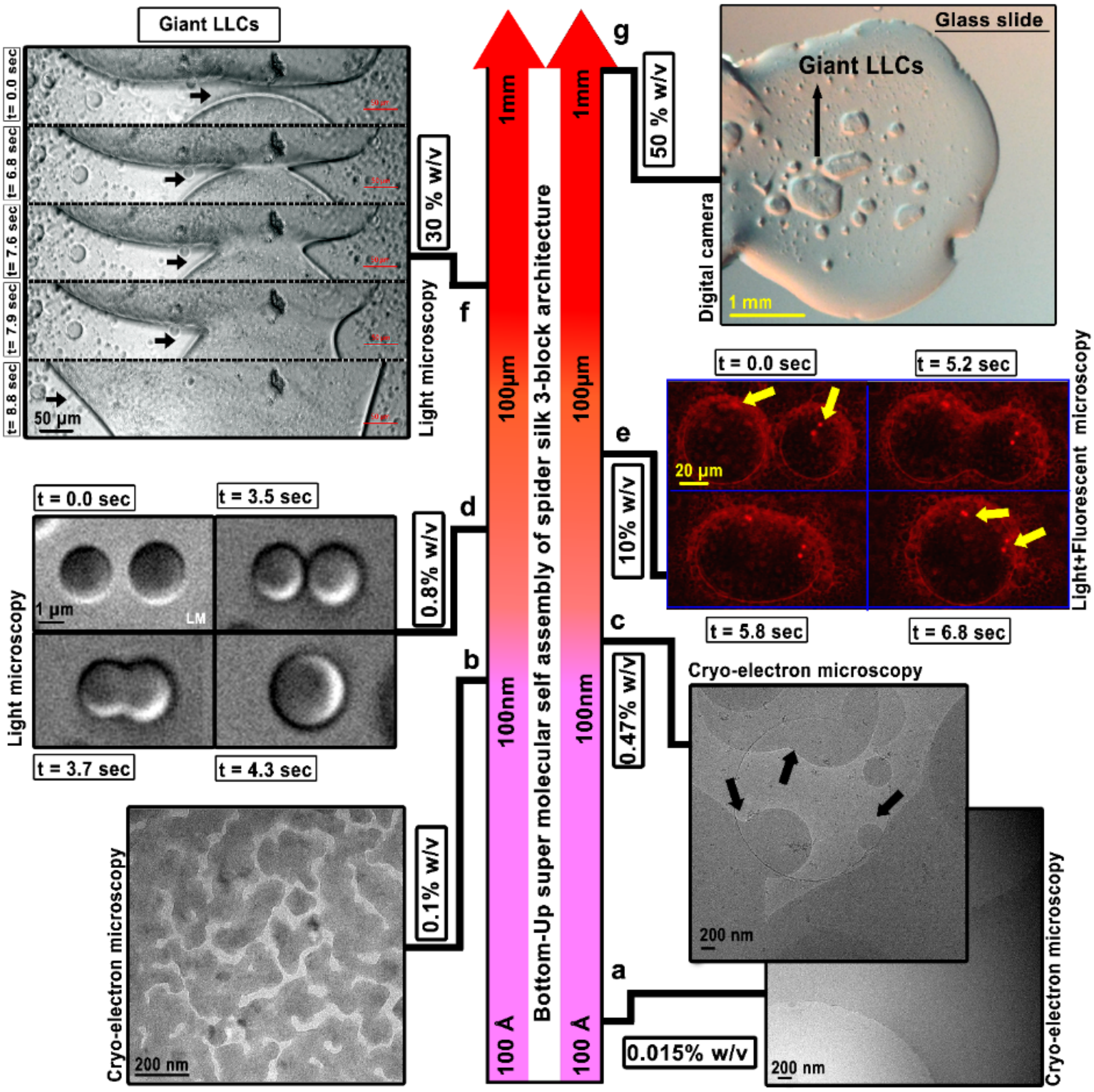


**Supplementary Figure 2.** LLC formation of the spidroin 3-block protein (CBM-eADF3-CBM) in a concentration dependent manner from nano- to millimeter length scales. (a) Cryo-transition electron microscopy (Cryo-TEM) image of a 0.015% w/v protein solution. At this concentration no coacervation can be seen (scale bar is 200 nm). (b) Cryo-TEM image of LLC assembly initiate through and intermediate stage in which disperse free monomeric proteins self-assembled into partially ordered island like clusters at overall concentration of about 0.1% w/v. Although the protein solution remains transparent to the naked eye clusters are formed that can be sedimented by centrifugal forces (scale

bar is 200 nm). (c) Cryo-TEM image of assembly of these initial clusters formed in the dense phase at the bottom of the centrifugal device at higher overall concentrations of 0.45-0.5% w/v, which eventually leads to formation of first nanometer size spherical/ellipsoid LLCs (scale bar is 200 nm) (Black arrows indicate some of these arrested coalescences of coacervates). (d) Bright field light microscopy images showing coalescence of micrometer-scale LLCs formed after increasing the overall concentration at different time points (scale bar is 1 µm). (e) Going beyond this critical threshold of 10% w/v enhanced the formation of increasingly large LLC droplets. Overlaid normal light and fluorescent microscopy images at different time points showing how two micron-size LLC droplets that had formed at > 10% w/v concentrations and contain each 2 encapsulated fluorescent particles (1 µm in diameter indicated with yellow arrows) fuse into one droplet (scale bar is 20 µm). (f and g) Images taken at different time points illustrating the coalescence events for two giant LLC droplets that are formed by increasing the concentration till >30% w/v (scale bar is 50 µm). Coalescence for different LLC sizes leads to a rapid exchange and rearrangements of internal content until they relax and assemble into micrometer sizes spherical LLCs. Such rapid relaxation times are expected for liquid compartment and coacervates with low interfacial surface energy. By calculating from the frame numbers we estimated that coalescence for droplets sizes bellow 100 µm happens at time scales of around hundreds of milliseconds and could reach to seconds for LLCs larger than 200 µm to fully fuse together.

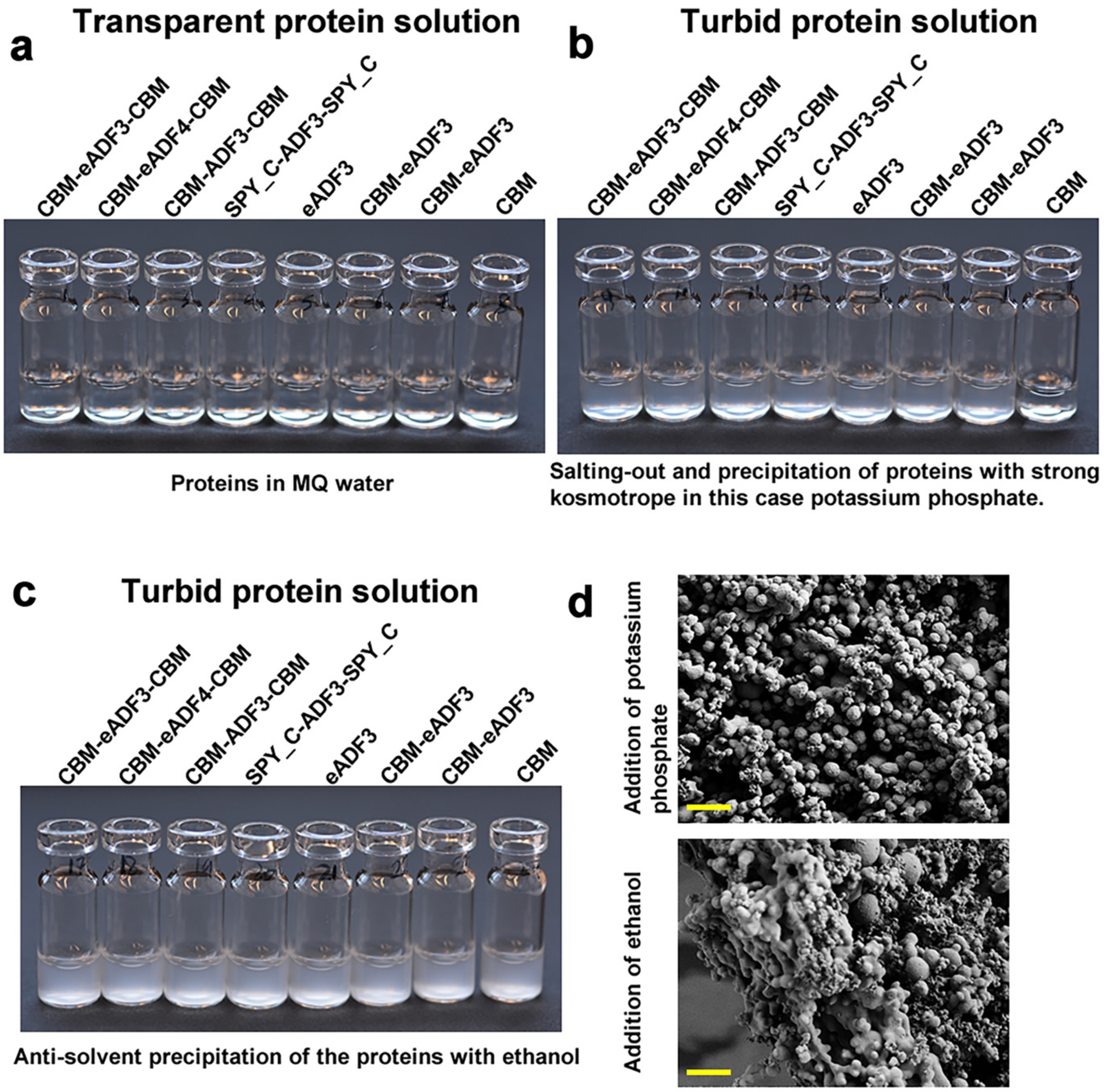


**Supplementary Figure 3**. Effect of kosmotropic salt and anti-solvent on solubility of the fusion proteins and formation of aggregates. (a) Fixed concentration of fusion proteins (0.1% w/v) in each tube. (b) Fusion proteins mixed with potassium phosphate (pH 7.4) at final w/v concentration of 0.05% to final molar concentration of 500 mM respectively. (c) Fusion proteins mixed with ethanol at final w/v concentration of 0.05% to final v/v concentration 48% respectively. (d) Scanning electron micrograph from precipitates of CBM-eADF3-CBM formed after addition of potassium phosphate (upper panel) and ethanol (lower panel) (scale bars are 1 µm).

The results indicate that the SLC formation in this study behave similarly as previously reported where anti-solvent precipitation [25] and kosmotropic salts [24,] have been used to form microspheres in the size of nano- to micrometers and used for protein purification by fractionation.

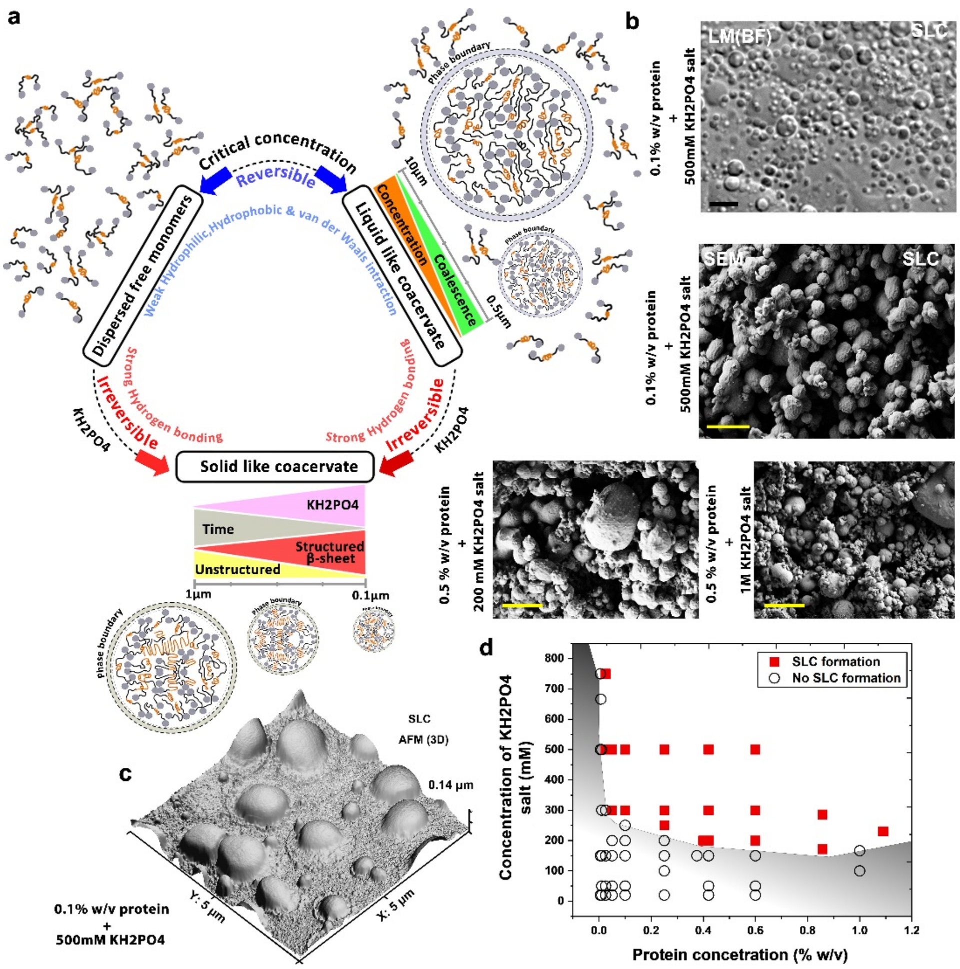


**Supplementary Figure 4.** (a) Schematic illustration of the processing pathways for LLC and SLC formation. When phosphate is indicated it was always added in the form of potassium phosphate ($KH_2PO_4$ at pH:7.4). (b) Bright field light microscopy of the salt induced SLC solution (scale bars are 20 µm) and scanning electron microscopy image of the same sample. CBM-eADF3-CBM mixed with potassium phosphate (pH 7.4) at final w/v concentration of 0.05% to final molar concentration of 500 mM respectively (scale bars are 10 µm). (c) Atomic force microscopy image of the SLCs from the same solution rendered in 3D. (d) SLC formation phase diagram of SLC formation for CBM-eADF3-CBM in potassium phosphate. Empty black circles indicate combinations in which no SLC formation could be observed whereas full red squares indicate combinations in which SLC observed with microscopy technique. The grey shading serves as a guide to the eye.

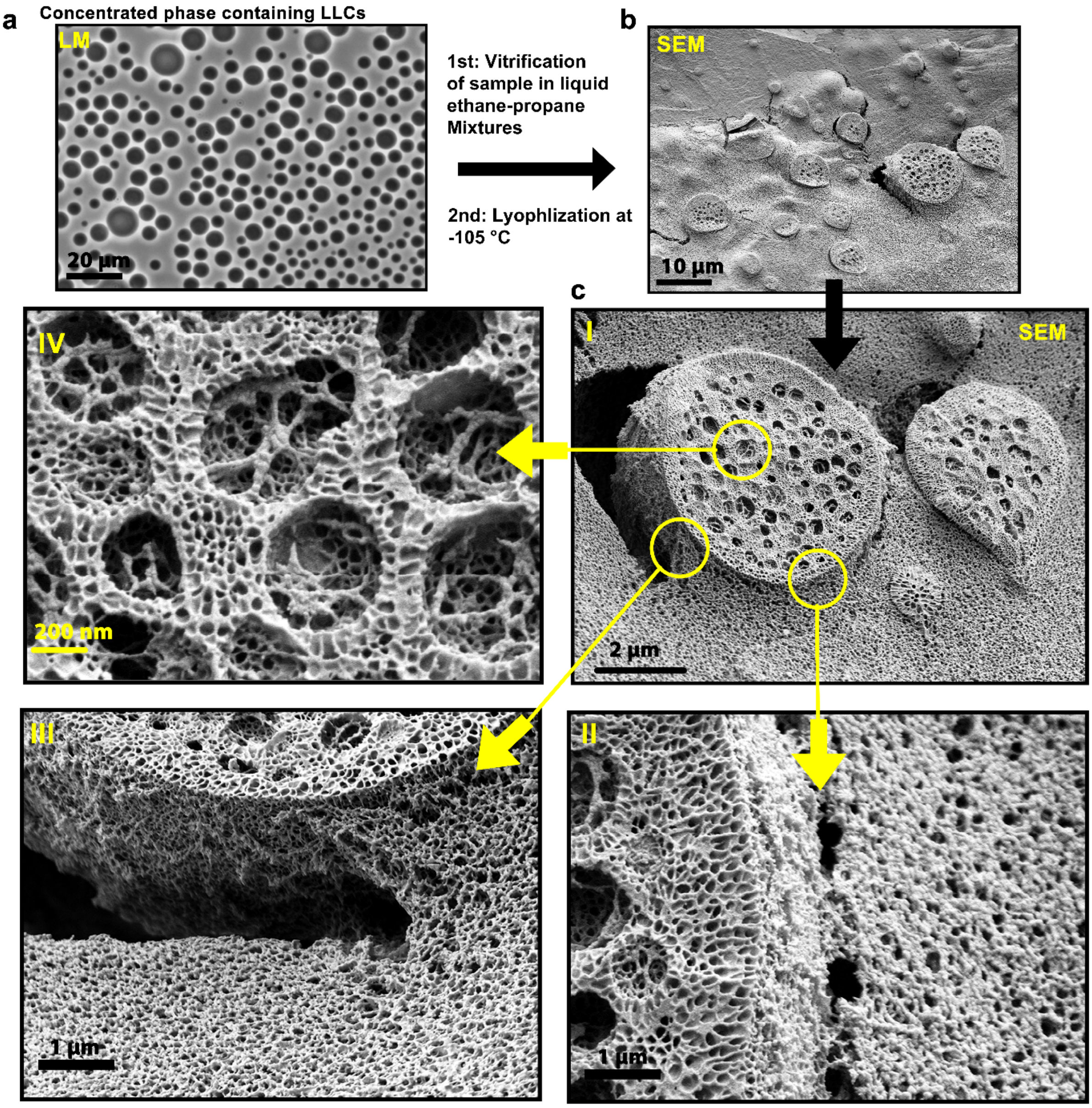


**Supplementary Figure 5.** (a) Bright field light microscopy image of the condensed phase containing the LLCs CBM-eADF3-CBM (scale bar 20 µm). (b) Scanning electron micrographs of same specimen arrested by vitrification of the solution in liquid ethane-propane mixture (50%:50%) at -180 °C followed by lyophilization. Probing the fractured specimen illustrates complex imprisonment of LLCs in continuous matrix of the free monomeric proteins (surrounding unstructured matrix) (scale bar 10 µm). (c) I, II and III) higher magnification images illustrating clearly the boundary between an LLC and the surrounding matrix which is formed from dispersed monomeric protein (scale bar 200 nm). IV) Internal structure of an LLC at high magnification illustrates extended fibrillar structural network (scale bar 200 nm).

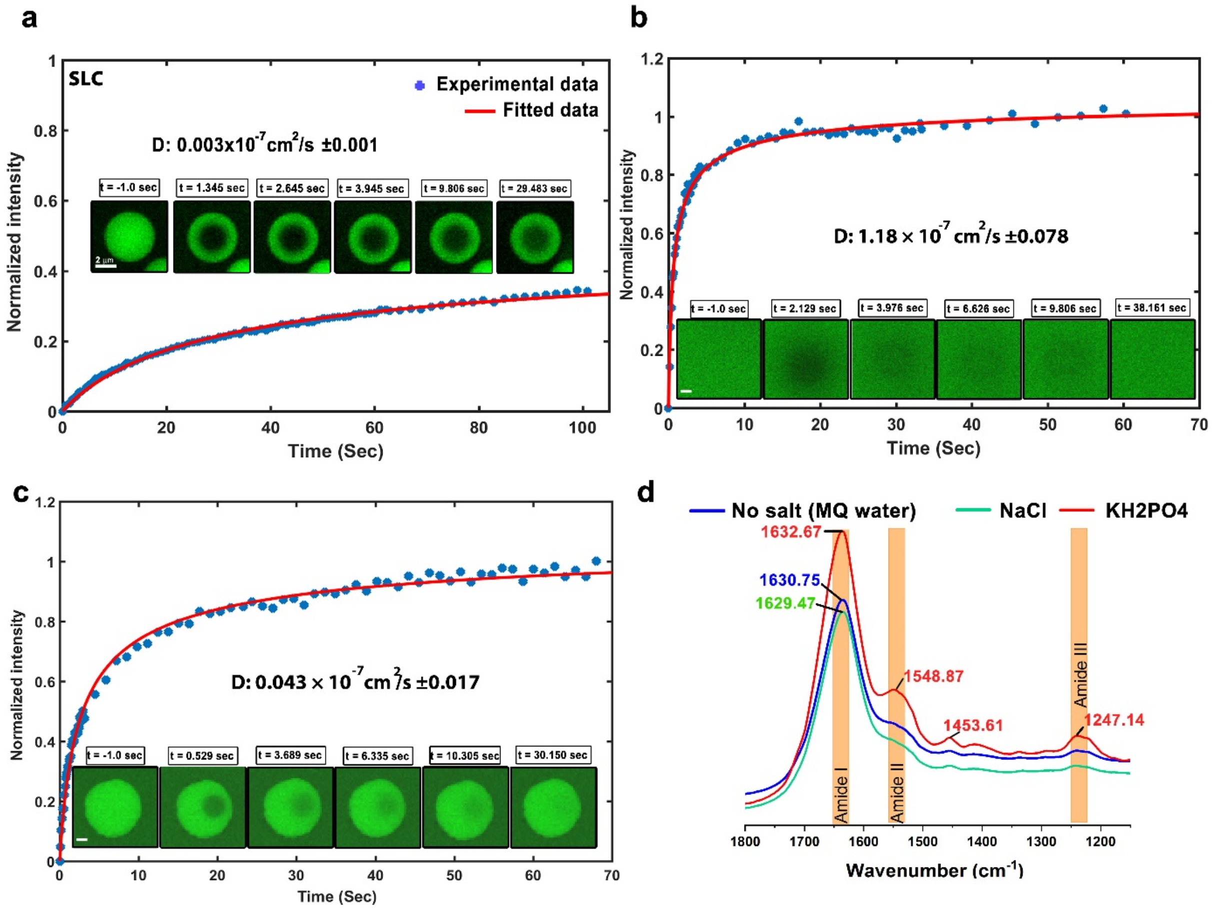


**Supplementary Figure 6.** (a) FRAP recovery of partially bleached CBM-eADF3CBM SLC (scale bar is 2µm (b) FRAP recovery for the surrounding dispersed monomeric proteins in the LLC solution of CBM-eADF3-CBM (scale bar is 2µm). (c) FRAP recovery of partially bleached CBM-eADF3 LLC (scale bar is 2µm). (d) FTIR spectra ranging from 1000 cm$^{-1}$ to 1700 cm$^{-1}$ (amide-I, II and III bands) illustrates intrinsic structural differences between LLC (blue line) and SLC (red line). Infrared spectrum of the LLCs showed a major amide I band at 1630 cm$^{-1}$ $^{v}$ (associated with bending vibration of C=O stretch), whereas SLCs showed the major amide I band at higher intensity with the absorbance peak at 1632 cm$^{-1}$ and prominent amide II band at 1549 cm$^{-1}$ ν (N-H). Together C=O and N-H associated with hydrogen bonding of the β-sheet/β-turn reach conformation in the SLCs. Furthermore, amide III band at 1247 cm$^{-1}$ ν (C-N) was also observed for the SLCs, corresponding predominantly to β-sheet.

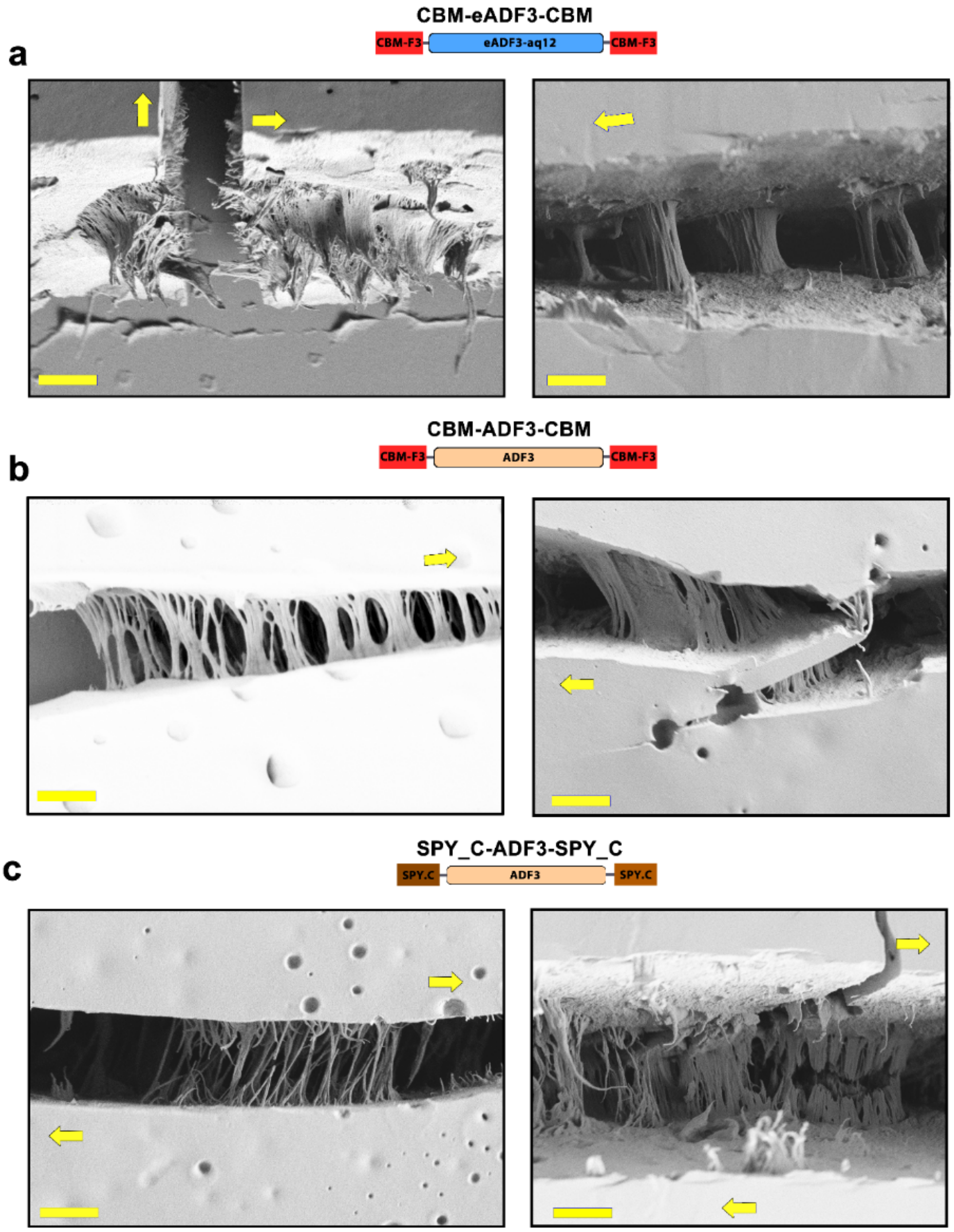


**Supplementary Figure 7.** SEM images of stretched dense LLC films of (a) CBM-eADF3-CBM, (b) CBM-ADF3-CBM and (c) SPY_C-ADF3-SPY_C. All films were prepared from the dense phase of protein solution from overall concentration of 2% w/v. Individual LLCs trapped in the film show high plastic deformation upon stretching, whereas the surroundings containing material containing monomeric proteins breaks as a brittle material (the scale bars in all the images are 2 µm).

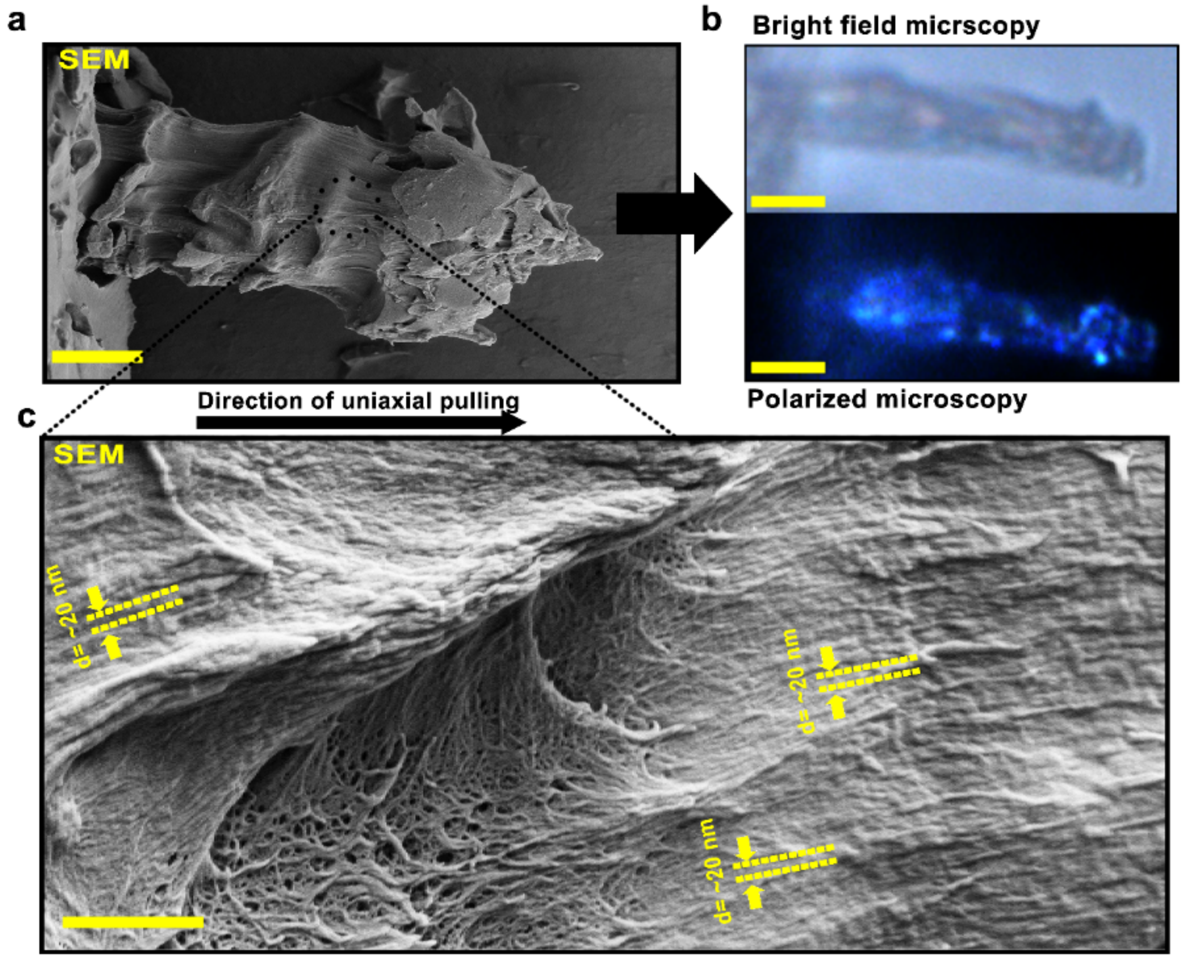


**Supplementary Figure 8.** Organization of the bundles of fibrils in the stretched film. (a) SEM micrograph of a single stretched LLC that was pulled out from the cracked area during stretching (scale bar is 1 µm). (b) Polarized microscopy of the same filament showing birefringence (scale bar is 2 µm in both panels). (c) High magnification SEM image from the surface of a stretched film illustrating highly aligned bundle of fibrils with the periodicity of 20 nm stretched in the direction of the extension (scale bar is 200 nm).

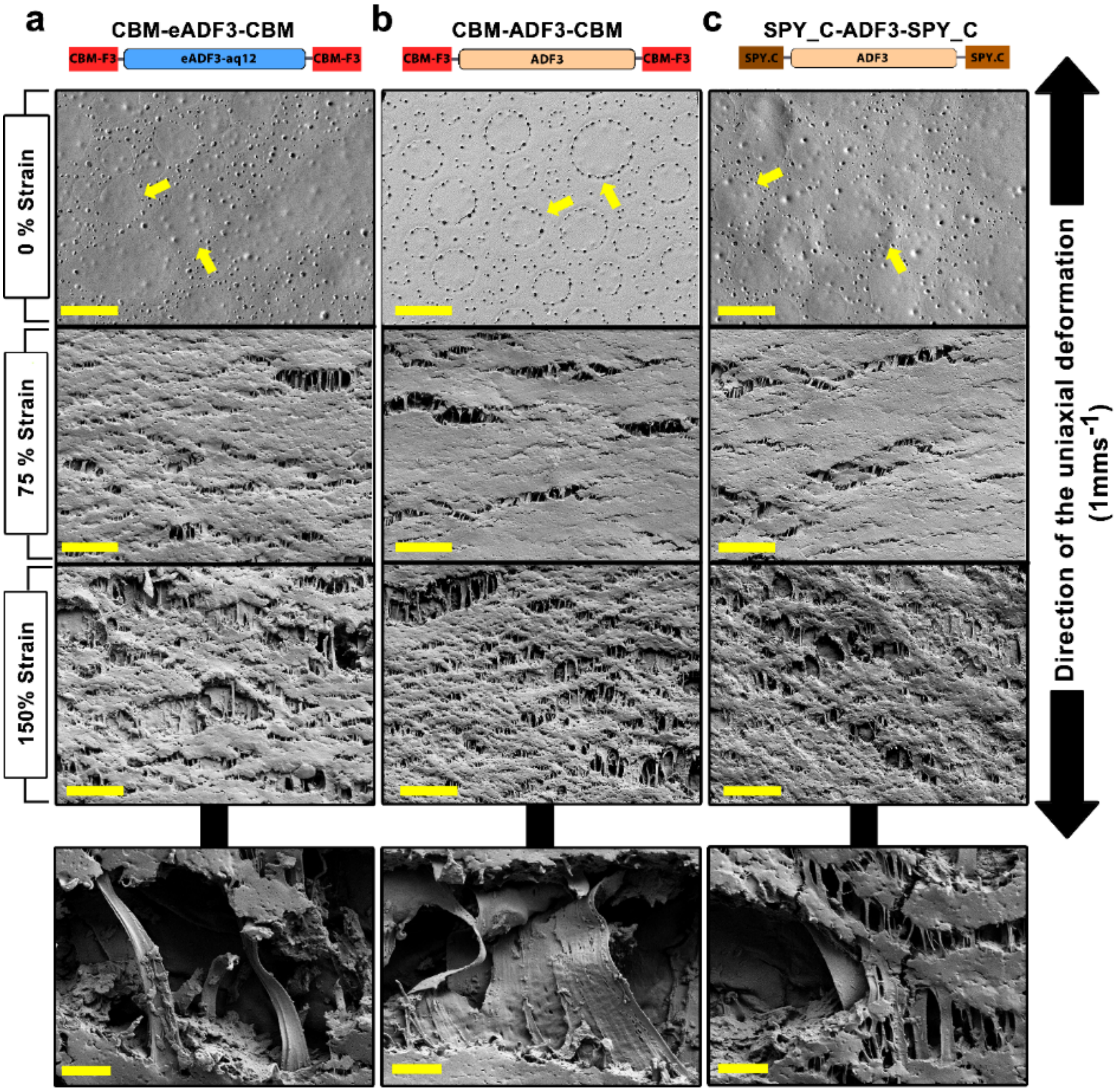


**Supplementary Figure 9.** Pulling of the semi-dried LLC phase results in the formation of long filaments with structural features from nano- to micrometer length scales upon mechanical stretching. A thin film casted from LLC containing dense phase of protein solution of (a) CBM-eADF3-CBM (b) and CBM-ADF3-CBM (c) SPY_C-ADF3-SPY_C LLCs at concentrations of 30% w/v. Yellow arrows indicate individual LLCs clustered within the film before mechanical stretching (0% strain). Upon straining the semi-dried film at 100% and 200%, LLCs are pulled out into long filaments and flat ribbons (scale bar is 20 µm for 0%, 100%, and 200% SEM images and 10 µm for all high magnification images of different constructs shown at the bottom panels).

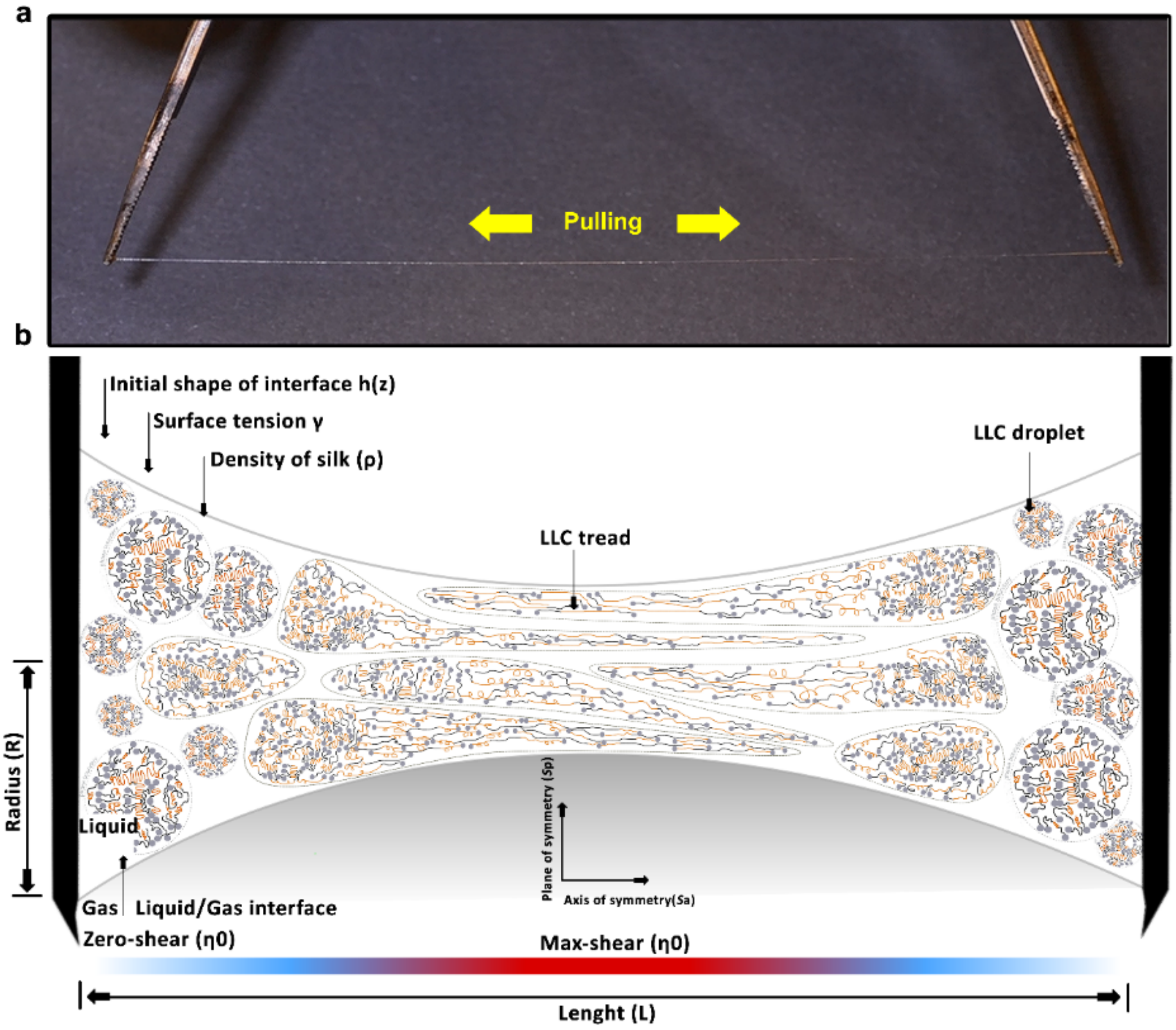


**Supplementary Figure 10. Fiber pulling.** (a) Single filament obtained by pulling a 10-15 µl droplet of highly concentrated (70-75% w/v) LLC between the tips of a pair of tweezers. Uniaxial pulling (approximately 1 mm $s^{-1}$) of the LLC solution leads to rapid formation of solid filaments in air. Simultaneous thinning and water evaporation throughout the length of the pulled filament facilitate transition of concentrated silk LLC solution into stable solid filaments. (b) Cartoon of how LLC droplets may be affected by the filament extension suggesting how dynamics and driving forces of formation are likely to rely on an intricate interplay of capillary, viscous, elastic, inertial and stretching forces. (components are not drawn to scale)

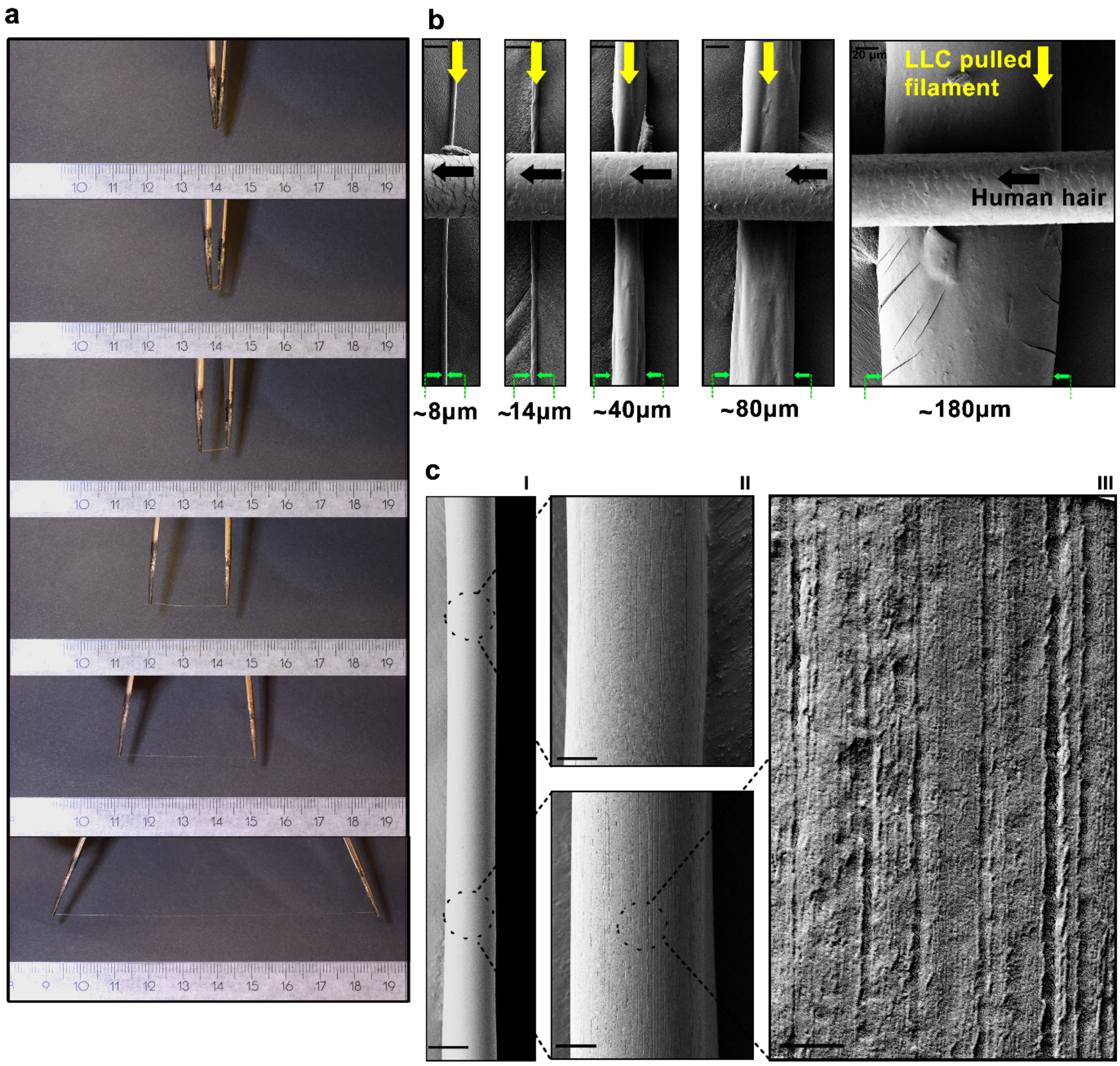


**Supplementary Figure 11.** (a) Rapid formation of filaments by extension of concentrated LLC condensed phase of CBM-eADF3-CBM. (b) Pulled filaments of different diameters ranging from 8 to 180 μm. To better illustrate the thickness in which filaments can be pulled, a human hair placed over them as a reference. (Note: filaments at larger diameter show large defects (many cracks), whereas thinner filaments show no defect which made them suitable for tensile measurement tests). (c) SEM images of a single pulled filament (scale bar 10 μm for C-I). High magnification images from the surface of the filaments illustrating molecular alignment (scale bars are 10 μm, 2 μm, and 500 nm for C-I, C-II, and C-III, respectively).

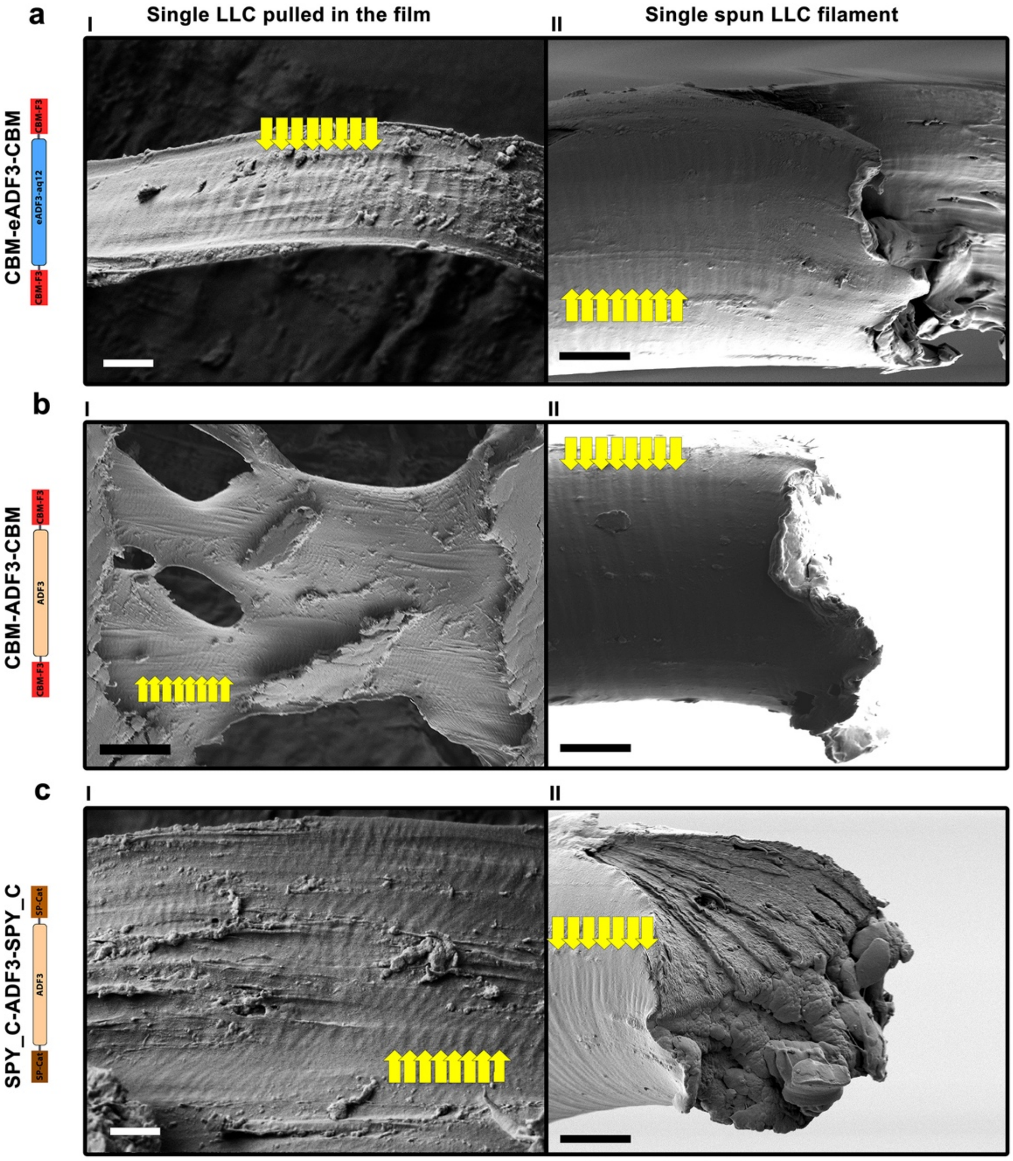


**Supplementary Figure 12.** (a-I, b-I and c-I) illustrate high magnification SEM micrographs from the surface of a filament formed by stretching of single LLC within the casted LLC condense films of CBM-eADF3-CBM, CBM-ADF3-CBM and SPY_C-ADF3-SPY_C. Parallel ring patterns can be seen crossing over oriented bundle of nanofibrils (scale bar is 500 nm for a-I and c-I and 1µm for the b-I). (a-II, b-II and c-II) illustrates the corresponding spun filament from the same constructs in which similar ring pattern can be seen on the surface of the pulled and fractured filaments (scale bar is 5 µm). Yellow arrows in both set of images are showing the ring pattern.

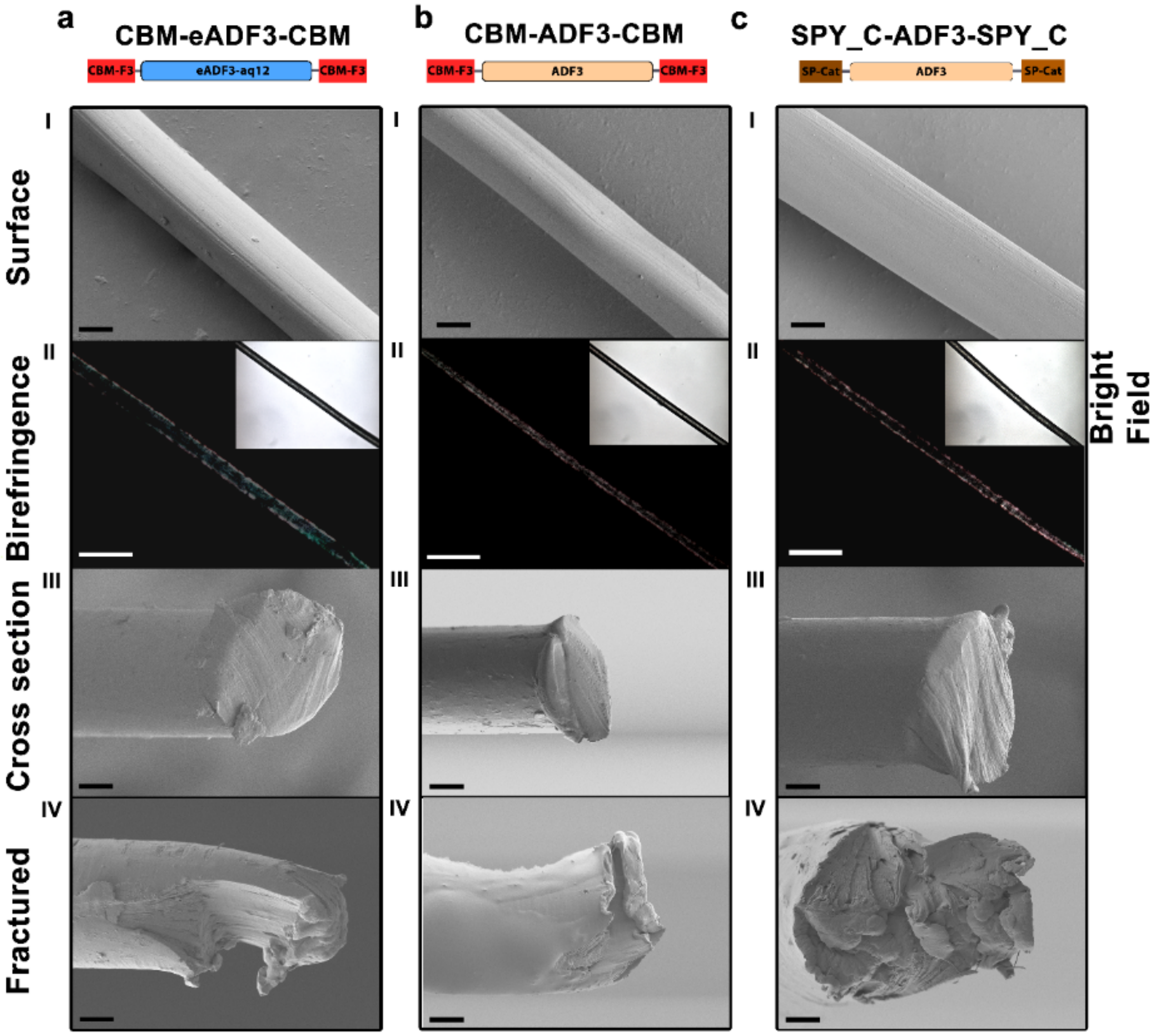


**Supplementary Figure 13.** (a-I, b-I and c-I) SEM images representing the surface morphology of the filaments for CBM-eADF3-CBM, CBM-ADF3-CBM and SPY_C-ADF3-SPY_C. (a-II, b-II and c-II) show polarized and light microscopy images of single filament placed between crossed polarizers at 45° with respect to the filament axis, illustrating the birefringence of the spun filaments for the corresponding constructs. (a-III, b-III and c-III) representing SEM micrographs of the cross sections of the filaments cut with razor blade. (a-IV, b-IV and c-IV) SEM images of fractured filaments for the corresponding constructs (scale bars are 2 µm).

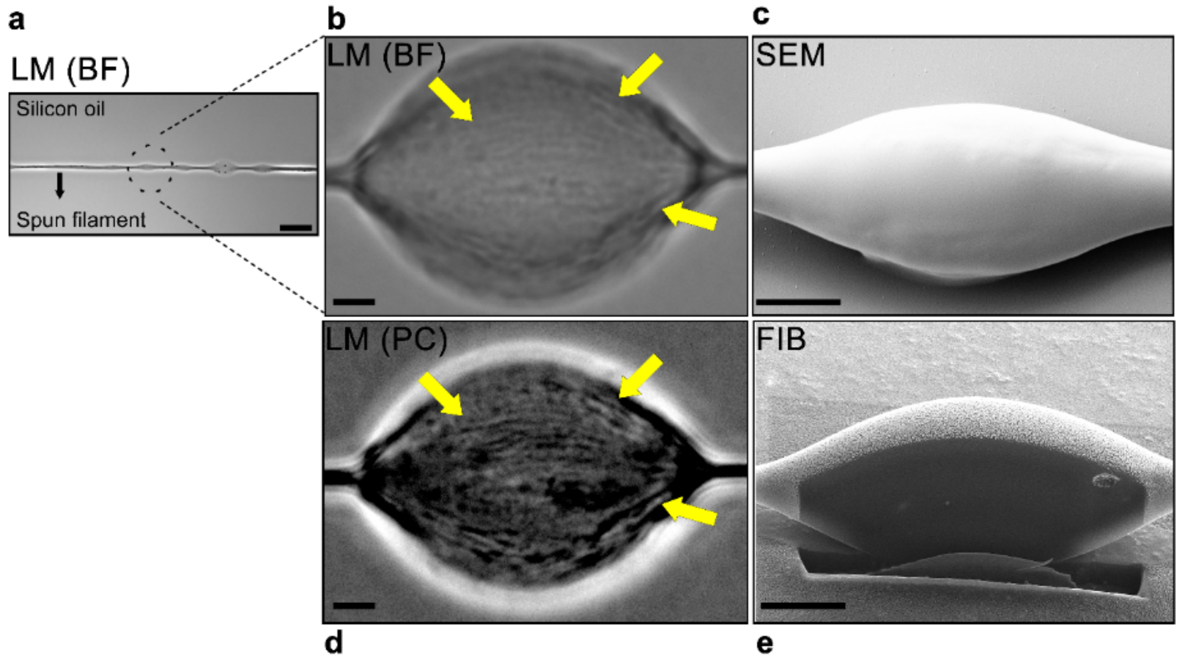


**Supplementary Figure 14.** Viscoelastic properties of the LLC solution occasionally lead to formation of beads on the surface of the pulled filaments. (a) Light microscopy (LM) Bright field (BF) image of such filament with multiple beads. In order to prevent drying of the specimen and providing chance to study structural characteristics of the beads in semi-dried state, freshly spun filament was placed on the surface of the glass slide and immediately covered with silicon oil (scale bar is 50 µm). (b) High magnification bright field and (c) phase contrast (PC) images of single and flattened bead. Yellow arrows indicate fibrils stretching from one side to the other side of the bead (scale bar is 5 µm). (d) SEM image of a bead representing the surface morphology (scale bar is 20 µm). (e) Focused ion beam milling of a bead, illustrating that internal structure of the bead become highly dense as it is air dried. Similar fibrillar structures could not be seen if the filaments were dried completely (scale bar is 20 µm).

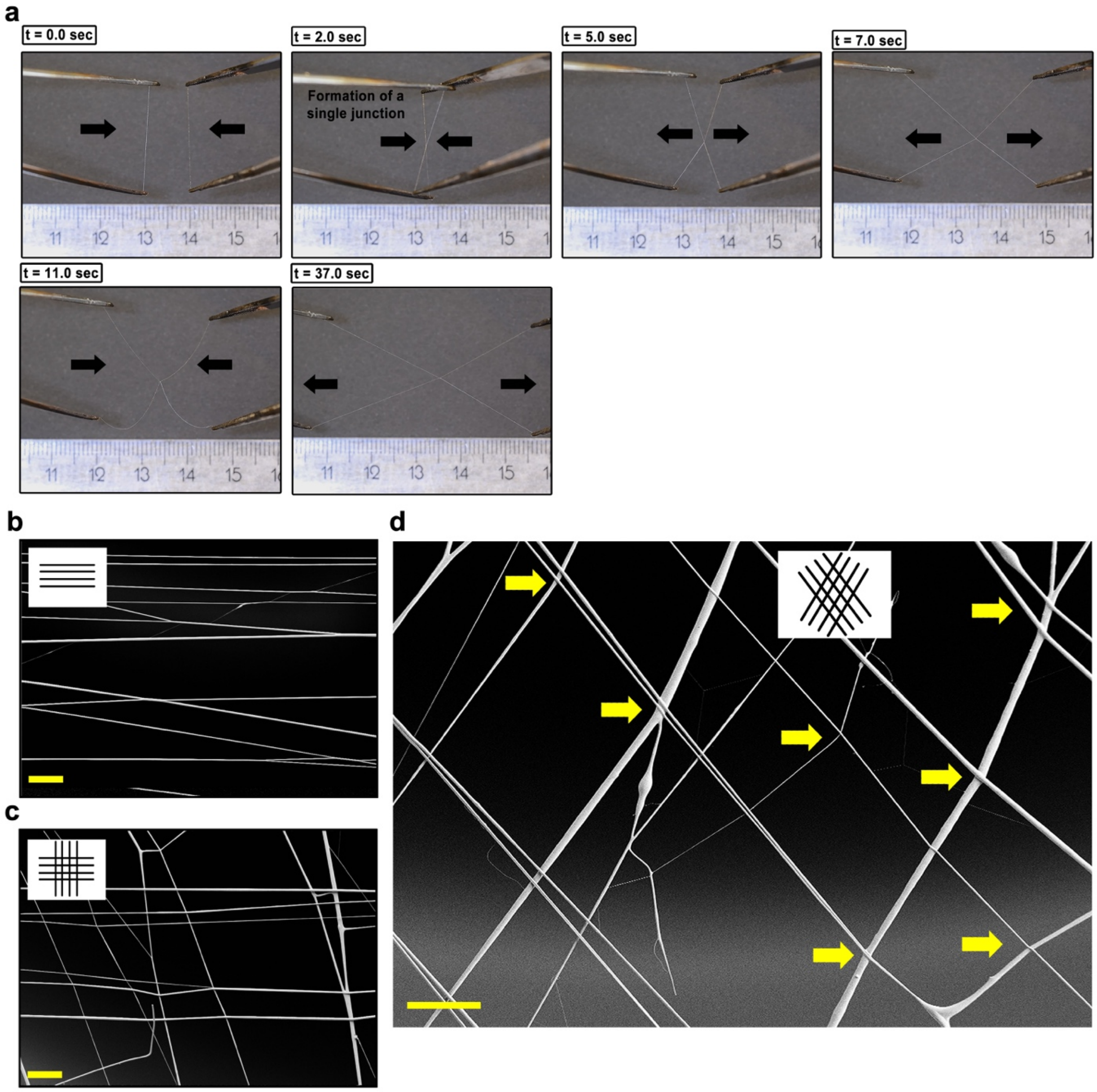


**Supplementary Figure 15. self-amalgamating characteristic of the freshly pulled filaments.** (a) Formation of a junction between two freshly pulled filaments at different time points. Black arrows represent direction of the movements. Before the formation of the junction (t = 0.0 seconds), during and after the formation of the junction (t = 2.0 seconds and t = 5.0 seconds). Repeating cycles of stretching and relaxation (t = 7.0 seconds, t = 11.0 second and t = 37.0 seconds). Junctions are strong enough to withstand the axial stretching. See also Supplementary Video 3. (b) Spun filaments horizontally placed in parallel to each other. (c) and (d) illustrate self-netted mesh like fabric made by overlaying multiple filaments across each other by taking advantage of self-amalgamating property of the freshly pulled fiber in wet state. (Some of the junctions are indicated with yellow arrows). The scale bars are 50 mm in all panels.

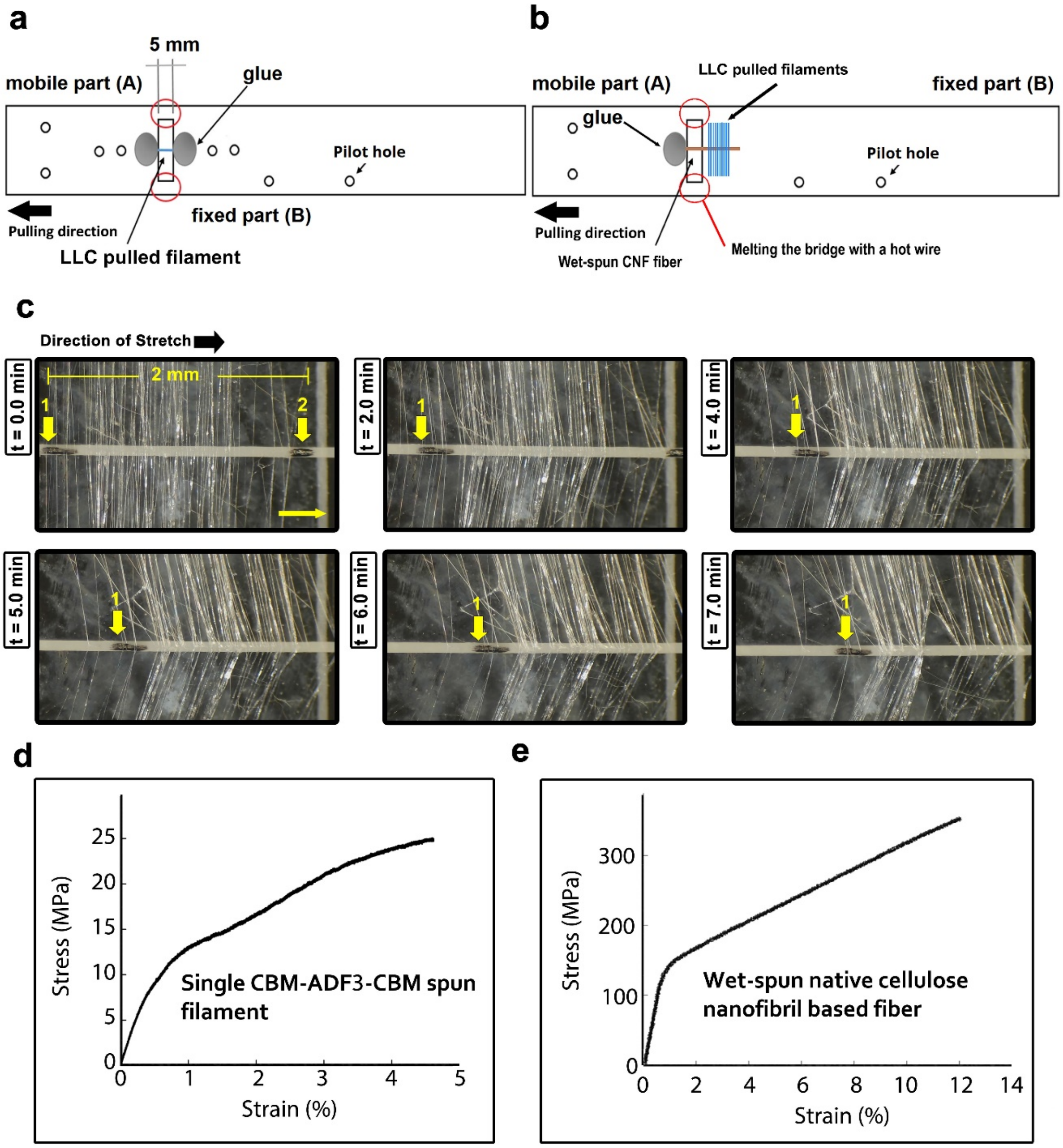


**Supplementary Figure 16.** (a) Custom-made sample holder for micro-mechanical measurements of a single filaments**.** (b) Custom-made sample holder for mechanical measurement tests for pyriform inspired adhesive attachment discs. (c) Snapshots taken during adhesive force measurement of pyriform inspired attachment disk. Single wet-spun cellulose nanofibril based fiber was hold down and attached by approximately seventy freshly spun filaments distributed and packed over 2 mm (Note: 2 mm area marked by two yellow arrows 1 and 2 in t=0.0, however arrow number 2 is not shown in further images as it moved out of field of view during adhesive force measurement). (d) and (e) Stress-strain curves for a single LLC pulled CBM-ADF3-CBM filament and wet-spun pure cellulose nanofibril, respectively, to show that wet-spun cellulose filaments are much stiffer in comparison to LLC pulled filaments. Hence, the wet-spun cellulose filament did not undergo plastic deformation or catastrophic

failure during the adhesive measurement, and the measured forces during the measurement were entirely generated from breaking and slippage of the LLC pulled filaments.

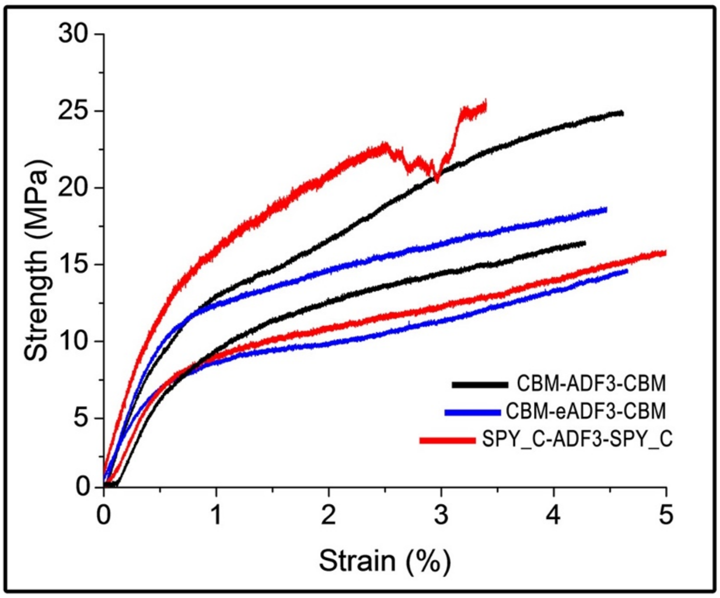


**Supplementary Figure 17.** Two representative stress-strain curves for LLC spun filaments of CBM-ADF3-CBM (black), CBM-eADF3-CBM (blue), and SPY_C-ADF3- SPY_C (red) constructs. Mean values and standard deviations are given in Supplementary Table 3.

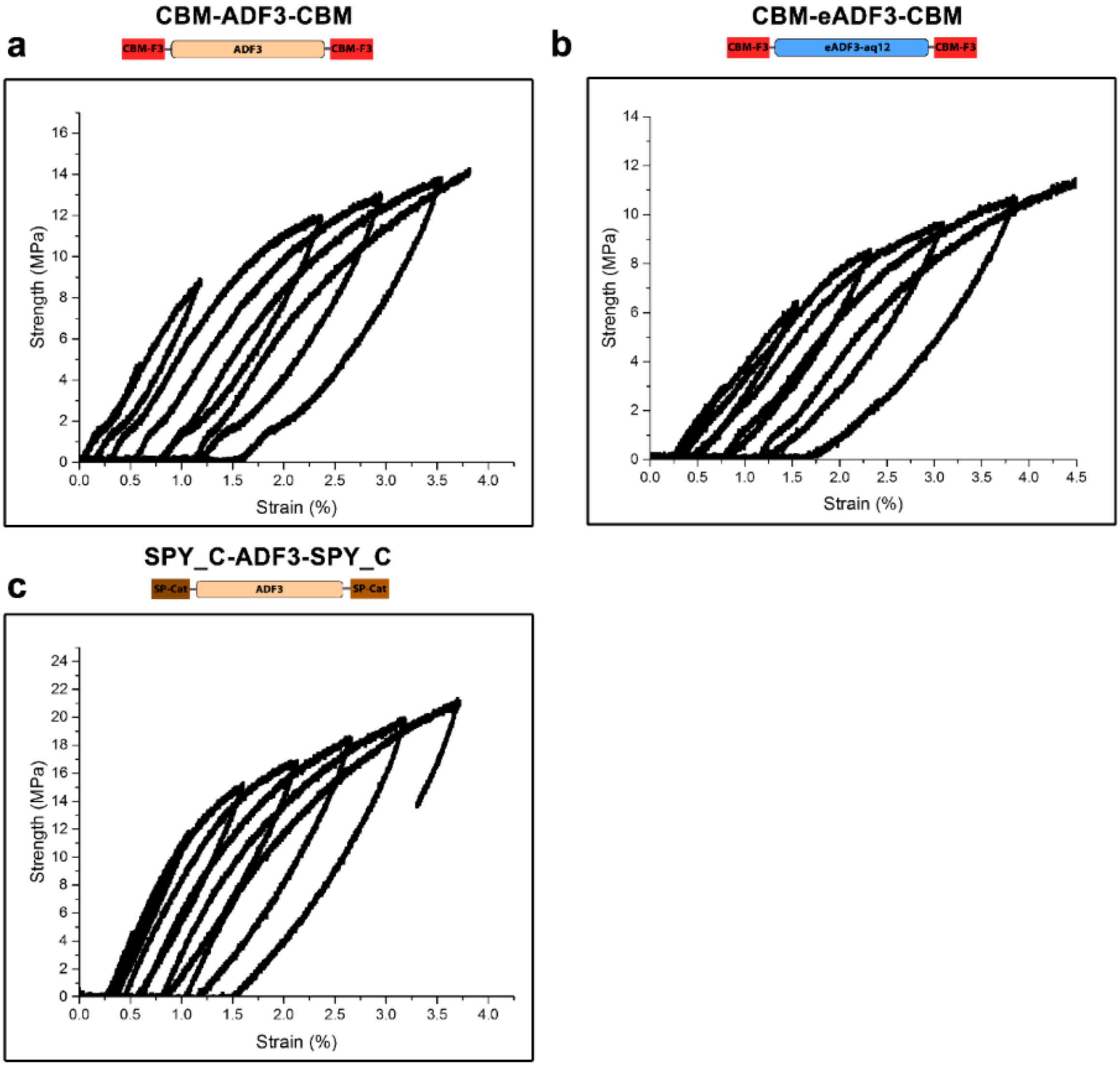


**Supplementary Figure 18.** Stress-strain curves from cyclic measurements in which filaments were allowed to relax for 5 min between loading and unloading cycles. (a), (b) and (c) illustrate cyclic tensile tests for CBM-ADF3-CBM, CBM-eADF3-CBM and SPY_C-ADF-SPY_C, respectively.

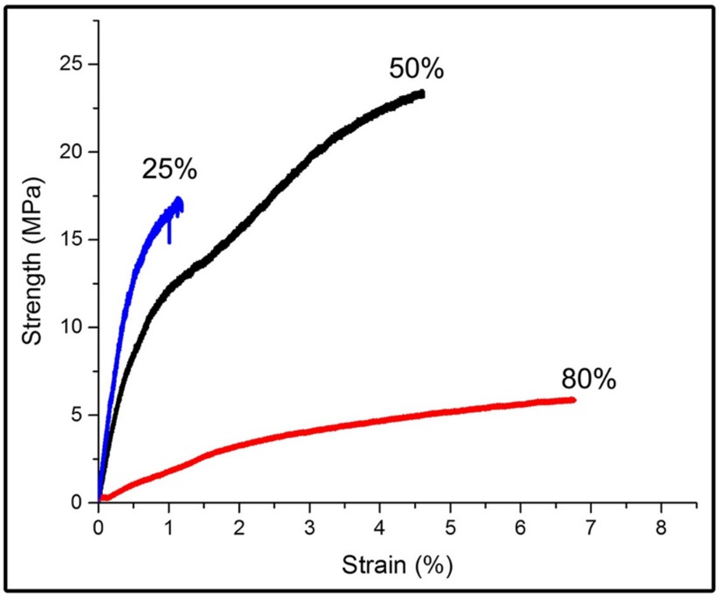


**Supplementary Figure 19.** Representative stress-strain curves for dried LLC spun filaments of CBM-ADF3-CBM at 25% (blue), 50% (black) and 80% (red) relative humidity (RH). Mean values and standard deviations are given in Supplementary Table 4.

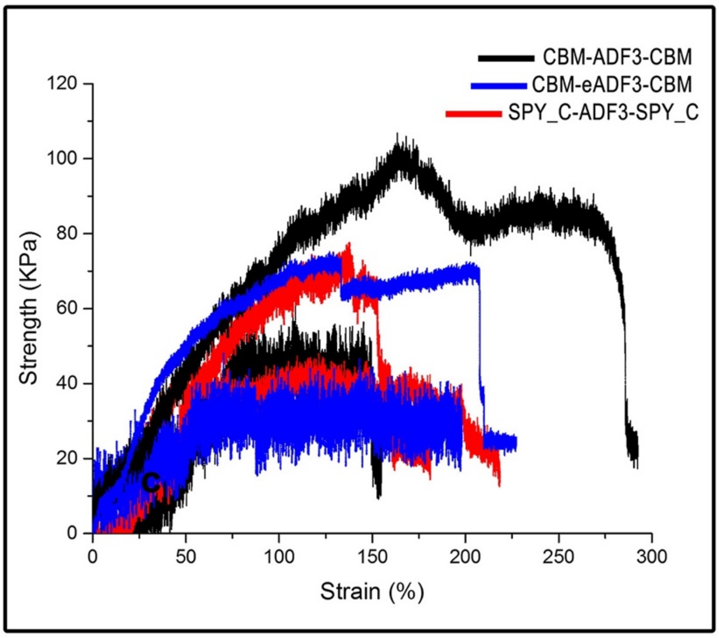


**Supplementary Figure 20.** Two representative stress-strain curves for semi-dried spun filaments of CBM-ADF3-CBM (black), CBM-eADF3-CBM (blue) and SPY_C-ADF3- SPY_C (red) constructs (Note: filaments were dried for 5 min prior to tensile measurement test). Mean values and standard deviations are given in Supplementary Table 3.

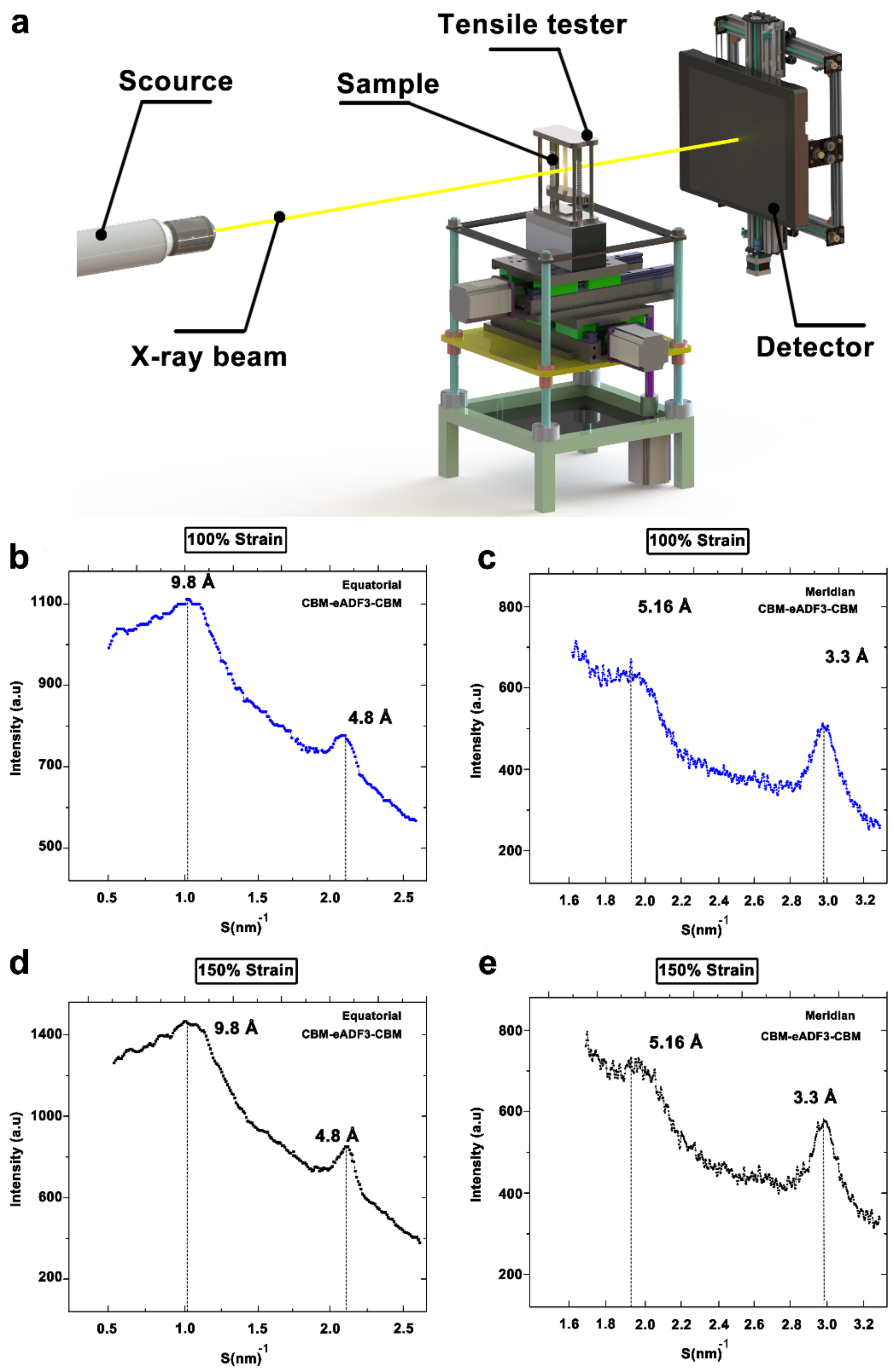


**Supplementary Figure 21.** (a) Schematic showing the beamline setup for *in situ* stretch-hold deformation using a tensile tester and simultaneous wide-angle x-ray scattering collection. One-dimensional meridian and equatorial profiles extracted from the two-dimensional wide angle X-ray diffraction pattern by integrating the intensity on a 10-pixel-thick rectangular strip for the spun CBM-eADF3-CBM (engineered sequence) filaments at 100% (b and c) and 150% (d and e) strains

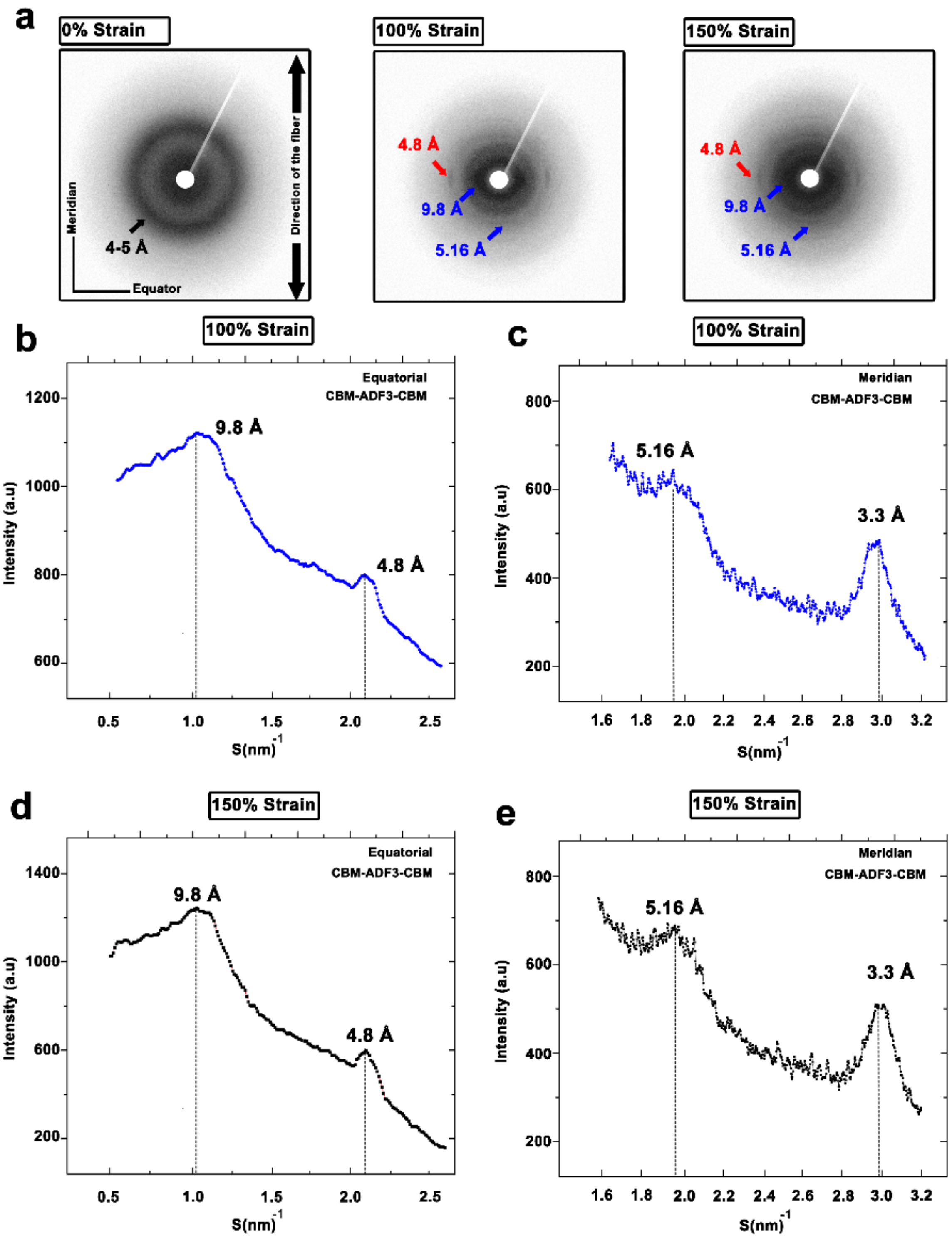


**Supplementary Figure 22.** (a) 2D wide angle X-ray (WAXS) diffraction pattern for a bundle of CBM-ADF3-CBM (wild-type sequence) spun filaments at 100% and 150% strains. One-dimensional meridian and equatorial profiles extracted from the two-dimensional pattern by integrating the intensity on a 10-pixel-thick rectangular strip for the spun CBM-ADF3-CBM filaments at 100% (b and c) and 150% (d and e) strains.

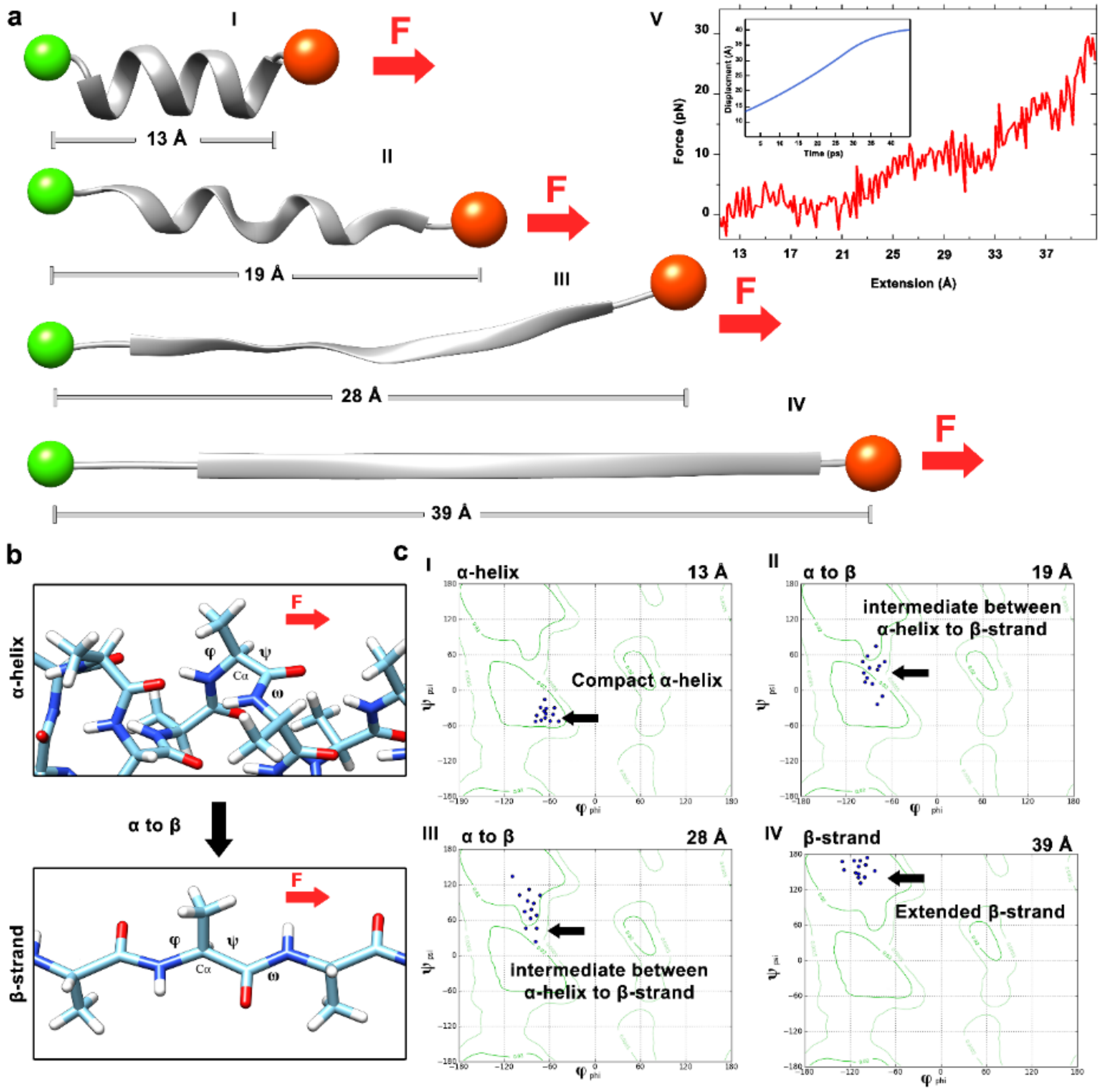


**Supplementary Figure 23.** Steered molecular dynamic simulation and effects of the pulling force applied to a single constructed model of polyAla block (lowest theoretical energy conformation of (GASAAAAAAGGY)) and the conformational transitions upon axial pulling in our study. (a) Snapshots of unfolding events taken from SMD simulation for a single α-helix showing, (I) the initial equilibrated polyAla model before stretching, (II and III) unfolding of the helix upon dissociation of hydrogen bonds and (IV) fully stretched β-strand conformation. The green sphere is the fixed atom, red sphere is the pulled atom, direction of the pulling force is shown with the red arrow and F stands for force. (V) Force versus extension profile of the single polyAla block stretched over the course of the simulation. (b) Illustrating existence of both possible conformations (α-helix and β-strand) for the polyAla block in more details in which backbone and side chains are shown in order to elucidate the positions of

dihedral angles (ϕ and ψ) between alanine residues. (c) Ramachandran plots calculated from four different trajectories throughout the course of the SMD simulation. The dihedral angles for α-helix (I) and β-strand (IV) are shown in which they are outlined (0.02) in two most favorable energy minima conformations. For the observed conformational transition (α-helix to β-strand) to occurs upon pulling, polyAla in α-helix has to go through (II and III) partially allowed and less energetically favorable region (0.0005). Many amino acids avoid conformations at these regions spatially at the outermost region of the 0.0005 largely due to steric conflicts. In the same line to this work, it has been illustrated that polyAla blocks within the repetitive region of spidroins in aqueous condition take α-helix conformation [3–12]. However, upon fiber spinning spidroin solution exhibits high shear forces from narrowing wall of spinning duct and most importantly pulling forces, which leads to conformational transition to β-sheets [24,26,3]. It has been illustrated that mechanical shear forces is sufficient to induce secondary structure interconversion [10,13,14], but other factors such as changes in the pH an ionic strength may also play a role during fiber spinning[15].

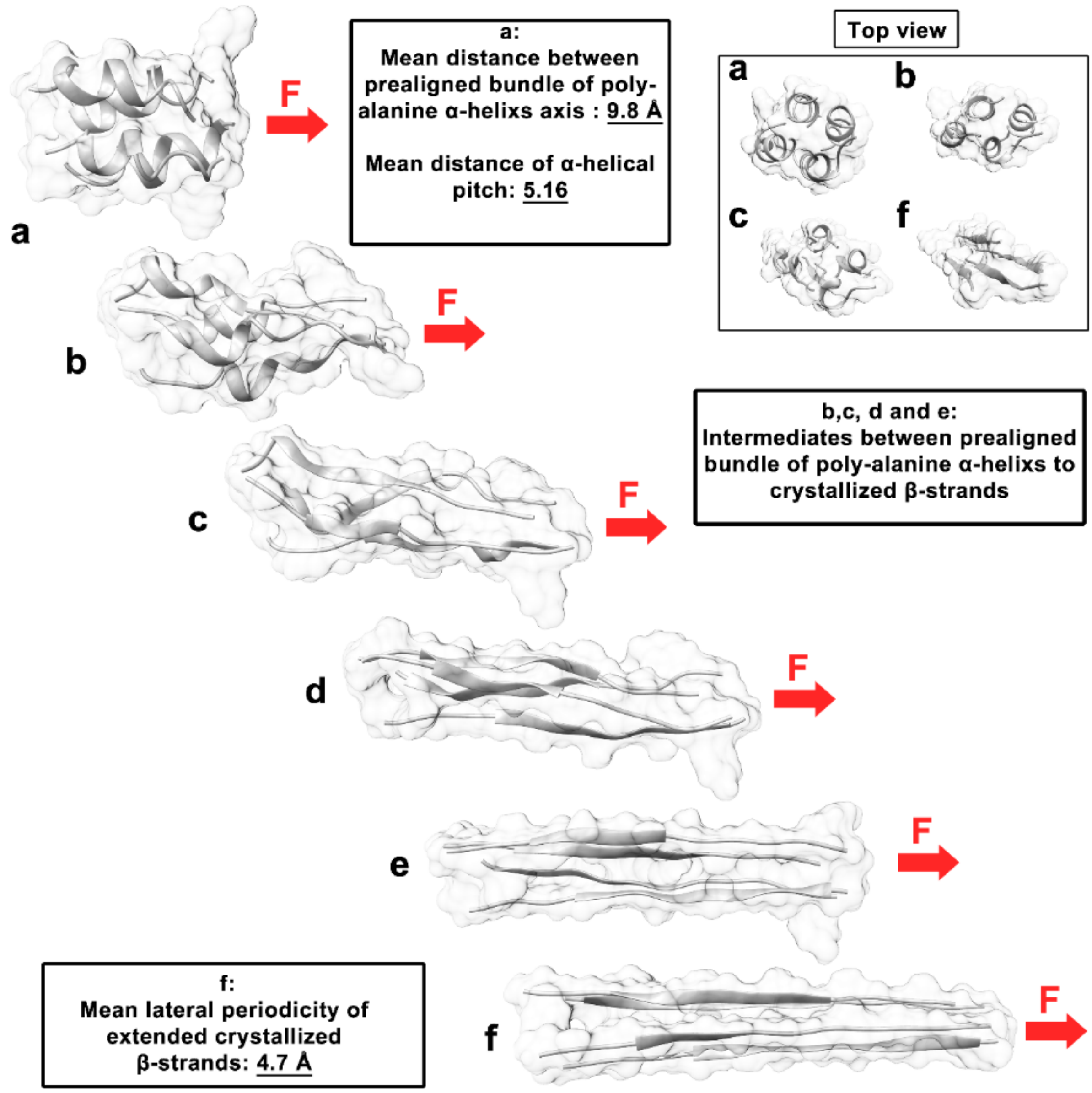


**Supplementary Figure 24.** Representative side and top views of four antiparallel α-helical bundles (a) before (b ,c, d and e) during and (f) after SMD simulation. WAXS analysis on filaments show two distinctive diffractions for the 100% and 150% post-stretched filaments. A broad peak with the highest intensity at 9.8 Å corresponds roughly to the mean distance between partially ordered α-helical axis[16,17] and lateral periodicity of polypeptide chains forming β-sheets, can be detected with the equatorial peak at 4.8 Å [18–22]. To validate the assignments for these two recognizable peaks from the experimental WAXS measurement we combined molecular modeling, steered molecular dynamic simulation (SMD) and scattering simulation (Fig. S27). We generated a bundle of four antiparallel α-helices (Fig. S26 a) and minimized the energy. The bundle was stable and alpha helices did not dissociate from one another through the course of the simulation. By measuring the inter α-helical

distance from 40 trajectory files we calculated the mean α-helical bundle spacing to be 9.8 Å, matching the observed experimental value. We did the same calculation for the fully stretched α-helical bundle, resembling if pre-ordered α-helices bundle were under mechanical stretch to form extended crystalized β-strands (Fig. S26 f). Calculating the mean lateral periodicity of the stretched model gave a value of 4.7 Å which is close to the experimental value of 4.8 Å.

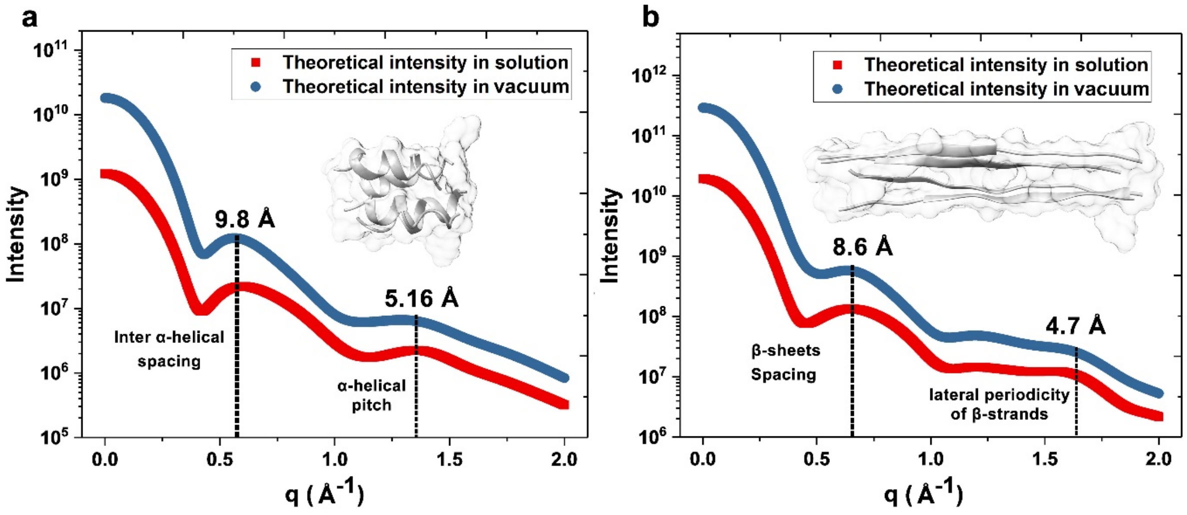


**Supplementary Figure 25.** Theoretical WAXS simulation of an α-helical bundle (a) and extended crystallized β-strands (b) calculated using the CRYSOL program. The coordinates derived from the MD simulation trajectories were used as input for the software.

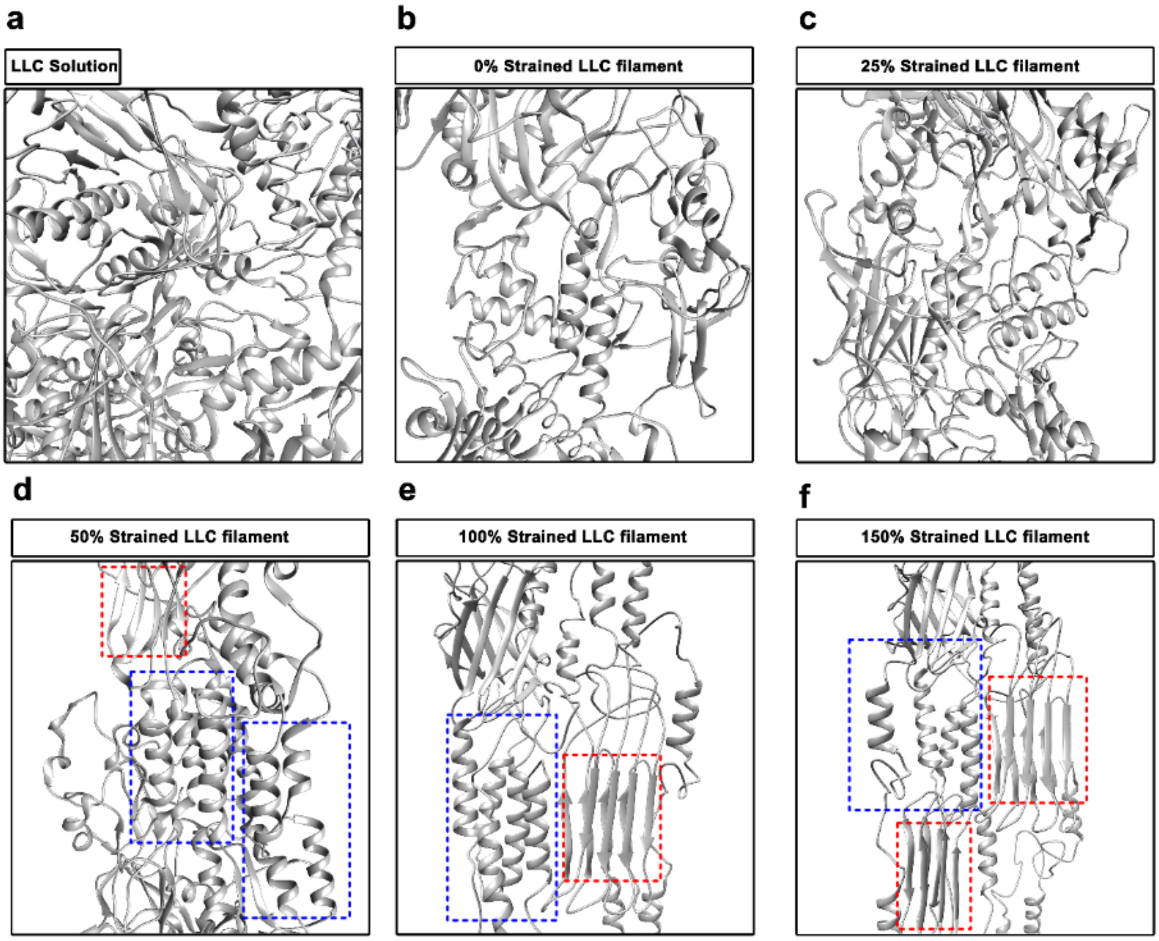


**Supplementary Figure 26.** Cartoon illustrating the possible effect of mechanical pulling forces and its effect on protein conformation from LLC dense solution to 150% post stretched filament. (a) Conformation of the proteins in LLC state, (b) Pre-formed filament, (c) 25% and (d) 50% post-stretched LLC filament, (e) 100% and (f) 150% post-stretched LLC filament. Blue boxes represent pre-aligned and packed bundle of α-helicals and red boxes represent extended crystalized β-strands. We hypothesize that filament formation of concentrated LLC (70-75% w/v) leads to multiple-sequential outcomes as follows: (1) inducing molecular orientation in the direction of the mechanical pulling, (2) polyAla block in α-helix conformation attain to close proximities and bundling to each other, increasing probability for stable cross β-sheet formation[5,23], (3) substantial reduction of bound water volume fraction and altering hydration patterns of the α-helices, and (4) global conformational transition of some of the α-helices to antiparallel β-sheets in the final dry filaments [14,24,25].

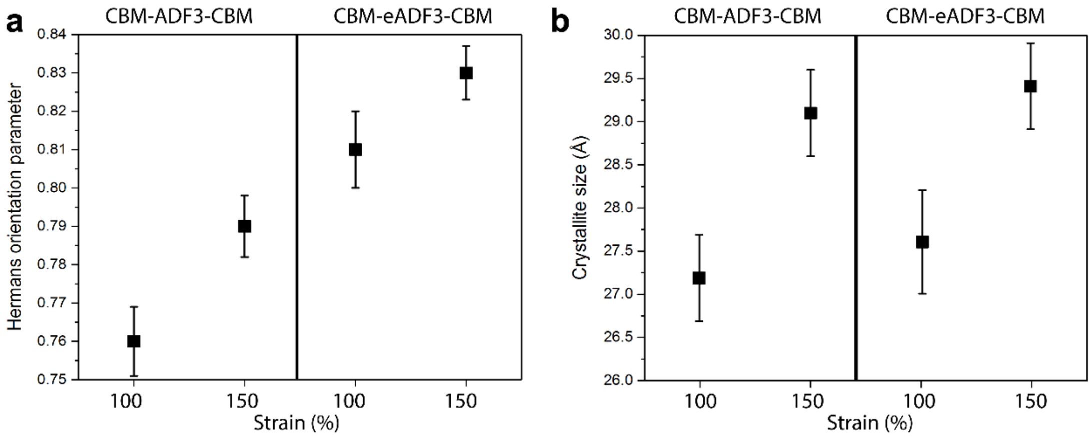


**Supplementary Figure 27.** (a) Calculated Hermans orientation parameters for CBM-ADF3-CBM and CBM-eADF3-CBM filaments at 100% and 150% strain. (b) Crystallite size calculated according to Scherrer equation for the same filaments respectively (Mean values and standard deviations are given in Supplementary Table 5) .

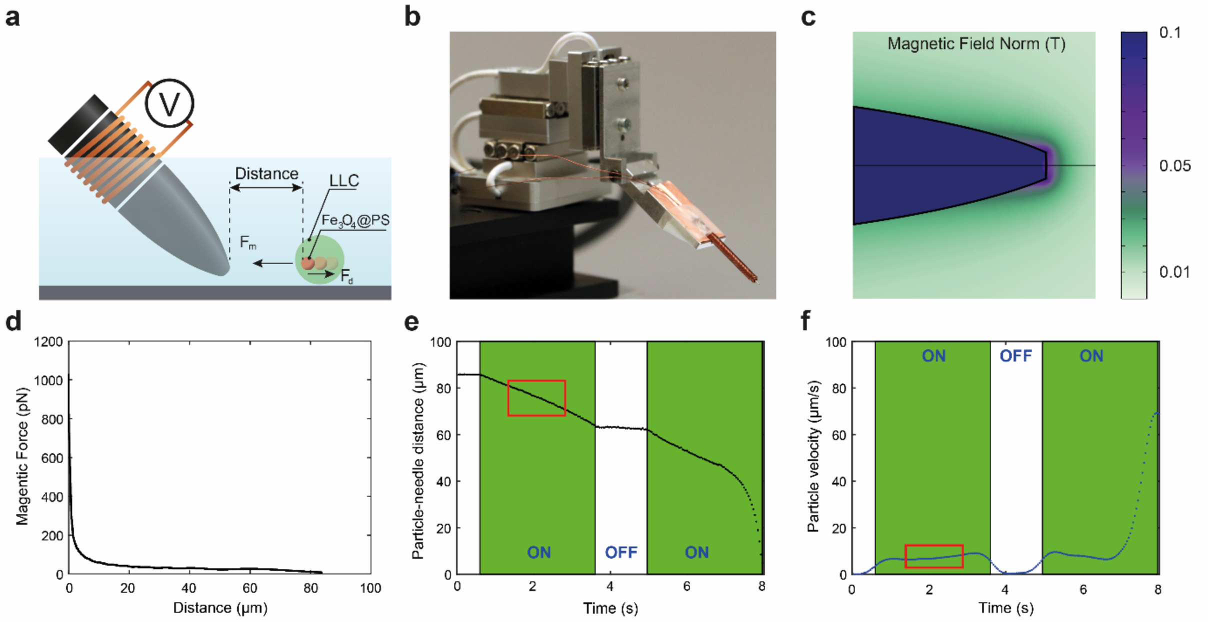


**Supplementary Figure 28.** Magnetophoretic viscosity estimation of LLC droplets. (a) Concept of magnetophoretic movement of encapsulated superparamagnetic microparticles within LLC droplets. Illustration not to scale. (b) Photograph of an electromagnetic needle attached to a manipulator with nanometer positioning resolution. (c) Numerical simulation results from Comsol Multiphisics 5.2 (Comsol Group, Sweden) of the magnetic field propagating out of the needle tip. (d) Calibrated dependency of the magnetic force over distance of a single microparticle for coil excitation current of 33 mA. (e) Particle-needle distance change during a magnetophoretic movement of a encapsulated particle within a protein droplet. (f) Particle-needle velocity during a magnetophoretic movement of a encapsulated particle within a protein droplet. The green colored quadrants in E and F denote the time when the current in the coil is ON, and the uncolored areas are denoting the time during which the current coil is OFF. The red rectangles denote a sample window extracted from the whole data for estimation of the protein droplet viscosity.

| **Supplementary Table 1. General information on the constructs used in the study.** | | | | | | | | |
|---|---|---|---|---|---|---|---|---|
| **Name in the text** | CBM-ADF3-CBM | CBM-eADF3-CBM | CBM-eADF4-CBM | SPY_C-ADF3-SPY_C | CBM-ADF3 | CBM-eADF3 | eADF3 | CBM |
| **Expression host** | *E. coli* | *E. coli* | *P.pastoris* | *E. coli* | *E. coli* | *E. coli* | *P.pastoris* | *E. coli* |
| **Molecular weight (Da)** | 82092.90 | 85288.34 | 84011.83 | 72296.37 | 66173.65 | 68013.50 | 47820.62 | 18226.91 |
| **Number of amino acids** | 863 | 889 | 914 | 783 | 719 | 715 | 541 | 163 |
| **Theoretical pI** | 5.77 | 5.77 | 4.46 | 5.05 | 6.28 | 6.01 | 7.00 | 6.20 |
| **LLC formation** | Yes | Yes | No | Yes | Yes | Yes | No | No |
| **Overall concentration for LLC assembly (% w/v)** | ~0.65 | ~0.85 | -Nil- | ~1.84 | ~8.18 | ~8.43 | -Nil- | -Nil- |
| **Fiber pulling** | Yes | Yes | No | Yes | No | No | No | No |
| **Aliphatic index** | 34.29 | 34.06 | 36.41 | 32.09 | 30.04 | 26.78 | 16.08 | 58.59 |
| **Grand average of hydropathicity (GRAVY)** | -0.721 | -0.780 | -0.441 | -0.721 | -0.741 | -0.872 | -1.005 | -0.638 |

| Supplementary Table 2. Summary of the mechanical properties of spun filaments (n=5) . | | | | | | |
|---|---|---|---|---|---|---|
| **Semi-dry filaments (50% RH)** | | | | | | |
| | Strength [MPa] | Extensibility [%] | Young's Modulus [GPa] | Toughness [MJ /m³] | Diameter (µm) | Sample size |
| **CBM-ADF3-CBM** | 0.078 ±0.033 | 225 ±106 | 0.16 ±0.003 | 7.4 x $10^{-5}$ ±3.0 x $10^{-5}$ | 99 ±20 | 5 |
| **CBM-eADF3-CBM** | 0.055 ±0.021 | 215 ±21.71 | 0.14 ±0.008 | 5.6 x $10^{-5}$ ±2.3 x $10^{-5}$ | 83 ±14 | 5 |
| **SPY_C-ADF3-SPY_C** | 0.048 ±0.018 | 200 ±42.28 | 0.15 ±0.002 | 6.1 x $10^{-5}$ ±26 x $10^{-5}$ | 91 ±17 | 5 |
| **Dried filaments (50% RH)** | | | | | | |
| | Strength [MPa] | Extensibility [%] | Young's Modulus [GPa] | Toughness [MJ /m³] | Diameter (µm) | Sample szie |
| **CBM-ADF3-CBM** | 16.65 ±8.81 | 4.16 ±1.57 | 0.762 ±0.361 | 0.719 ±0.156 | 30 ±7 | 5 |
| **CBM-eADF3-CBM** | 16.14 ±5.25 | 4.12 ±1.7 | 0.743 ±0.425 | 0.745 ±0.173 | 27±6 | 5 |
| **SPY_C-ADF3-SPY_C** | 16.4.77 ±5.42 | 4.57 ±2 | 0.835 ±0.529 | 0.662 ±0.177 | 23 ±110 | 5 |

| Supplementary Table 3. Summary of the mechanical properties of dried CBM-ADF3-CBM filaments measured at 25% and 80% relative humidity (n=5). | | | | | | |
|---|---|---|---|---|---|---|
| **CBM-ADF3-CBM** | | | | | | |
| | Strength [MPa] | Extensibility [%] | Young's Modulus [GPa] | Toughness [MJ /m³] | Diameter (µm) | Sample size |
| **25% relative humidity** | 11.23 ±6.43 | 0.8 ±0.28 | 1.3 ±0.6 | 0.08 ±0.02 | 22 ±8 | 5 |
| **80% relative humidity** | 4.6 ±3.74 | 6.5 ±1.95 | 0.13 ±0.05 | 0.221 ±0.09 | 25 ±4 | 5 |

| Supplementary Table 4. Calculated crystallite size and Hermans orientation parameters for CBM-eADF3-CBM and CBM-ADF3-CBM air pulled filaments. | | | |
|---|---|---|---|
| **Hermans orientation parameters** | | **Crystallite size** | |
| **100%** | **150%** | **100%** | **150%** |
| **CBM-eADF3-CBM** | | | |
| 0.79 ±0.086 | 0.83 ±0.067 | 27.6 ±0.5 | 29.4 ±0.4 |
| **CBM-ADF3-CBM** | | | |
| 0.76 ±0.083 | 0.81 ±0.069 | 27.2 ±0.6 | 29.1 ±0.5 |

## SUPPLEMENTARY METHODS

### Cloning, expression and purification in *Escherichia coli*

*E. coli* strain XL1B (New England Biolabs 5-alpha F'Iq Competent *E. coli* (F´ *proA*$^+$*B*$^+$*lacI*$^q$ *Δ(lacZ)M15 zzf::Tn10* (Tet$^R$) */ fhu A2Δ(argF-lacZ)U169 phoA glnV44 Φ80Δ(lacZ)M15 gyrA96 recA1 relA1 endA1 thi-1 hsdR17*)) was used for cloning purposes. Luria-Bertani (LB) medium and LB-agar plates were used for growth overnight at 37 °C with following antibiotics when appropriate: kanamycin (50 µg/ml), ampicillin (100 µg/ml). The resulting constructs were then transformed into *E. coli* strain BL21 (F-*ompT hsdS*B (rB-mB-) *gal dcm* (DE3)) (ThermoFisher Scientific) or BL21 T7 express (*fhuA2 lacZ::T7 gene1 [lon] ompT gal sulA11 R(mcr-73::miniTn10*--Tet$^S$*)2 [dcm] R(zgb-210::Tn10*--Tet$^S$*) endA1 Δ(mcrC-mrr)114::IS10*) (New England Biolabs) for expression. In general EnPresso B medium (BioSilta, Oulu, Finland) technology or MagicMedia™ *E. coli* expression medium (Thermo Fisher Scientific) was used for all the expressions according to manufacturer's protocol with some changes. In short, 1 colony from freshly grown overnight LB-plates was picked and cultured in 2 ml LB-media supplemented with kanamycin (50µg/ml) for 3-4 hours at 37 °C with shaking at 250 rpm . Cultivation was performed in 50 ml in 500 ml *Erlenmeyer* flask with first dose of glucose-releasing biocatalyst (0.4 U $L^{-1}$) and inoculation with 2 ml pre-culture. Flasks were sealed with air-permeable membranes and incubated at 250 rpm and 30 °C. After 18 hours of cultivation, second dose of biocatalyst (0.8 U $L^{-1}$), booster nutrients and 0.5 mM Isopropyl β-D-1-thiogalactopyranoside (Sigma-Aldrich) was added to the culture to induce protein expression. For the expression in MagicMedia 1 colony from freshly grown overnight plates (LB-Agar+kanamycin (50 µg/ml)) was picked and cultured in 20 ml of LB supplemented with kanamycin (50 µg/ml) and grown for 6-8 hours at 37 °C and 250 rpm. Flasks were sealed with air-permeable membranes. Entire 20 ml of precultured cells were inoculated in 500 ml of LB supplemented with kanamycin (50 µg/ml) and grown overnight at 30 °C and 250 rpm. 100 ml of overnight pre-culture was inoculated in 1 liter of MagicMedia™ with added component B and incubated at 30 °C and 250 rpm. Cultures in both Enpresso B medium and MagicMedia were harvested after 15-24 hours by centrifugation at 16,000 ×*g*, 15min, 4°C. Cell pellets were suspended in the final volume of 5 mL of Lysis Buffer (20mM 4-(2-hydroxyethyl)-1-piperazineethanesulfonic acid, pH 7.5, 5 mM $Mgcl_2$, 200 mM NaCl, 20 mM Imidazole (Sigma-Aldrich), 0.5 mg $ml^{-1}$ lysozyme (Chicken egg white, Sigma-Aldrich), 0.01 mg $ml^{-1}$ DNase I (Bovine pancreas, Sigma-Aldrich) and 1 × SIGMAFAST protease inhibitor cocktail (EDTA-free for every 1 gram of cells by incubating at 4°C on a roller shaker for 2 hours. Cell suspensions were then sonicated (Qsonica 500) on ice using 20% amplitude input, for 3 minute and 5 second intervals with tapered Microtip 1/8'' (3.2 mm) Ø. Alteratively, cells were homogenized by high

pressure cell homogenizer (AVESTIN-Emulsiflex-C3). Cell debris was collected by centrifugation at 16,000 ×*g*, 15 min, 4°C followed by purification using either immobilized metal affinity chromatography (IMAC) or heat fractionation. IMAC was performed using HisTrap FF crude columns (GE Healthcare Life Sciences) connected to an ÄKTA-Pure fast protein liquid chromatography system (Binding buffer containing 500mM NaCl, 20mM imidazole, pH 7.4 and Elution buffer containing 500mM NaCl, 500mM imidazole, pH 7.4 were used unless otherwise stated). Alternatively, lysed supernatant was heated to 70-75 °C for 30 min and then centrifuged at 16,000 ×*g* for 80 min at 4 °C in order to fractionate the thermostable proteins of interest into supernatant. Desalting, buffer exchange and concentrating of the samples carried out with Econo-Pac 10DG desalting prepacked gravity flow columns (Bio-Rad). Samples were then flash-frozen in liquid nitrogen and stored at -80 °C. Proteins were analyzed by standard sodium dodecyl sulfate-polyacrylamide gel electrophoresis (SDS-PAGE) using 5% stacking and 9% running gels. SDS-PAGEs were stained with Coomassie Blue R-250 (CBR) stain. Roughly 30 µg of proteins were loaded in the lanes.

**Cloning, expression and purification in *Pichia pastoris***

To constructs eADF3 alone, codon optimized synthetic genes for expression in *Pichia pastoris* were ordered from Geneart. DNA sequence encoding engineered recombinant spider silk protein based on the sequence of ADF4 from *Araneus diadematus* fibroin (eADF4) [26–28] in frame with N- and C-terminal CBMs was also codon optimized for expression in *Pichia pastoris*, ordered as an intact piece from GeneArt gene synthesis (ThermoFisher Scientific) and named CBM-eADF3-CBM. Both eADF3 and CBM-eADF4-CBM were then inserted into the pPICZ-α (Invitrogen) expression vectors. Products were then transformed into Top10 *E. coli* cells (ThermoFisher Scientific) and selected on zeocin low salt LB plates. The clones containing the assembled plasmid were verified by restriction enzyme analysis with XhoI/NotI and visualization in agarose gel. The expression vector was linearized with PmeI (New England Biolabs) and transformed into the X33 *Pichia pastoris* strain and the transformants were selected for on zeocin plates according to the manufacturer’s instruction (Invitrogen). These strains and a negative control, empty X33 strain, were cultivated in 250 ml flasks with 30 ml of growth medium at 30°C, shaking at 250 rpm. All three strains were first cultivated overnight in BMGY medium in order to achieve a high cell density. The cells were harvested by centrifugation (1,000 ×*g*, 5 min) after the density reached an $OD_{600}$ value of 3-6. The protein production was initiated by diluting the cells to $OD_{600}$=1.0 in 250 ml of BMMY medium in 2 liter flasks, adding protease inhibitors chymostatin and pepstatin A, and adding methanol daily to a final concentration of 2%. Culture samples were collected and analyzed on Criterion (4-20%) SDS PAGE gels (Bio-Rad) with Gelcode Blue Coomassie stain (ThermoFisher Scientific). Additionally, immunoblotting was done using a mouse anti-His tag antibody (Trend Pharmatech P-205) and detected via near infrared fluorescence with a goat anti-

mouse IgG IRDye 680R (Li-cor #926-68070) secondary antibody and scanned at 700 nm with the Odyssey CLx (LI-COR Biosciences). One liter of culture supernatant was collected after 27 hours, the cells were filtered away from the culture supernatant, and PSMF protease inhibitor was added to the supernatant to a final concentration of 0.5 mM. The culture supernatant was then used for purification of the silk proteins using either immobilized metal affinity chromatography (IMAC) or heat fractionation. IMAC was performed using HisTrap FF crude columns (GE Healthcare life Science) connected to a ÄKTA-Pure fast protein liquid chromatography system (Always binding buffer containing 500mM NaCl, 20mM imidazole, pH 7.4 and Elution buffer containing 500mM NaCl, 500mM imidazole, pH 7.4 were used unless otherwise stated).

**Cryo-transmission electron microscopy (Cryo-TEM)**

High-resolution transmission cryo-electron microscopy imaging was carried out using JEM-3200Fsc field emission microscope (JEOL) operated at 300 kV in bright-field mode with Omega-Zero-loss energy filter with a 20 eV slit. The micrographs were acquired with ULTRASCAN GATAN 4,000 CCD camera using digital micrograph software (GATAN). The specimen temperature was maintained at −187 °C. Prior to use 200 mesh carbon (CFT200-Cu) or Quantifoil 3.5/1 holey carbon copper grids with 3.5 µm holes were cleaned using Gatan Solarus 9500 plasma cleaner and assembled in FEI Vitrobor humidity chamber (100% humidity). 3 µl of samples were then applied on grid with blotting time of 1 second and vitrified in 1:1 mixture of liquid propane and ethane (-180 °C). Grids were handled under liquid nitrogen until transferred into microscope.

**3D reconstruction of SLC**

Before sample deposition TEM grids were dipped in ligand-coated gold nano-particle (11-mercapto-1-undecanol) solution (diameter: 2–10 nm) for image alignment. High-resolution cryo-electron tomographic tilt series were collected using SERIALEM-software package between tilt angles of ±69°. For fine image alignment, markers were detected automatically and cropped manually using IMOD. To increase signal to noise ratio images were binned twice and maximum entropy method used for 3D reconstruction. UCSF CHIMERA package was used to visualize and analyze the tomograms.

**Vitrification of the LLCs for characterization of the internal structures**

In order to preserve the soft structures of the LLCs in hydrated state we adapted the same technique widely used in preparation of biological and soft matters for cryo-transmission electron microscopy in which samples are vitrified in a mixture of propane and ethane (-180°C) at the rate of $\sim 10^6$ °C/s in order for the specimen to be fixed in vitreous state. One droplet (3 µl) of the condensed phase

containing LLCs was plunged and vitrified into a 1:1 mixture of liquid ethane (-180 °C). Samples were then handled under liquid nitrogen and transferred into a FreeZone 4.5 Liter Cascade Benchtop Freeze Dry Systems equipped with collector cooling chamber at -105 °C.

**Focused ion beam**

Dual beam FIB/SEM (FEI HELIOS) apparatus (Nanofabrication center, Micronova, Finland) was used for sectioning and imaging the pulled filaments and also beads formed on filaments. Milling was done using accelerated Gallium ions ($Ga^{+}$) at 30 kV with normal current conditions of 10, 100, 300, and 1000 pA. In order to avoid charging of the samples surface, samples were sputtered with 2 nm platinum coating and during operation electrons were accelerated to the target using low voltage of 1 to 1.5 kV.

**Atomic force microscopy (AFM)**

To study the morphology of SLC coacervates formed by addition of 500 mM $KH_2PO_4$, a SLC solution was first washed three times with Milli-Q water and then diluted 1 : 100 with Milli-Q water. A 10 µL aliquot was spread on a freshly cleaved mica surface. Samples were allowed to dry over 24 hours at ambient temperature and humidity before the imaging. A Veeco dimension 5000 AFM instrument was used and images were recorded in tapping mode in air with scan rates of 0.8–1 Hz with a FASTSCAN-B cantilever.

**Solid like coacervation (SLC)**

Fusion proteins mixed with potassium phosphate (pH 7.4) at final w/v concentration of 0.05% to final molar concentration of 500mM respectively to induce SLC coacervates. To study the phase diagram different concentrations of CBM-eADF3-CBM constructs and potassium phosphate (pH 7.4) were tested.

**Preparation of the electromagnetic needle**

An electromagnetic needle consisting of a copper wire (AWG 34) coiled around a sewing needle (martensitic stainless steel) in four layers with overall resistance of ~2 Ω. The needle was glued on a v-grooved aluminum holder with double-sided tape. The holder was fixed to an adapter, which was attached to a (3 DOFs) robotic nanopositioner (SLC1720, SmarAct GmbH, Germany) mounted on a standard inverted microscope (Axio Vert.A1, Zeiss, Germany).

**Calibration of the magnetic force produced by the electromagnetic needle**

The procedure for calibration of the magnetic force from the electromagnetic needle was done according to Kollmannsberger and Fabry and Lee[29,30]. The electromagnetic needle was mounted on an

inverted microscope (Axio Vert.A1, Zeiss, Germany) with objective lens (20×0.35 Ph1). Bright field microscopy was used throughout the experiment. The magnetic force was calibrated by tracking the velocity of red fluorescent and superparamagnetic $Fe_3O_4$@Polysterene microparticles (Microparticles GmbH, Germany) immersed in 80% glycerol solution. The magnetic force $\boldsymbol{F_m}$ causes the particle to move within the immersion solution. During the movement the magnetic force is opposed by the hydrodynamic drag $\boldsymbol{F_{hd}}$. The motion equation of a microparticle can be expressed as in formula (2), where $m$ is the mass and $\ddot{x}$ is the acceleration (second derivative of the position $x$ in respect to the needle). However, the inertial force can be dismissed ($m = 0$) since it is more than an order of magnitude lower than the magnetic force and the hydrodynamic drag. Hence equation (3) can be expressed as equation (4) , where $\eta_g = 6.14\ 10 - 2\ Pa \cdot s$ is the viscocity of 80% glycerol at 22 °C ,[30] $d = 4.5\ \pm 0.01$ is the particle diameter, and $v$ is the particle velocity. The trajectories of the particles were recorded with high-speed camera (Phantom Miro 310, Vision Research Inc., USA) with acquisition rate of 3000 frames per second. Particle tracking algorithm was based on Hough Transform [REF] and implemented within MATLAB (MathWorks, USA). The position data along each axis was smoothed with smoothing spline ($R^2 \geq 0.99$) followed by derivation, i.e. obtaining $v_x$ and $v_y$. Consequently, the magnitude of the velocity vector computed according to equation (5) giving the magnetic force-distance dependency.

(2)

$$\boldsymbol{m\ddot{x} = F_m - F_{hd}}$$

(3)

$$\boldsymbol{F_m = F_{hd} = 3\pi\eta_g dv}$$

(4)

$$|\boldsymbol{v}| = \sqrt{\boldsymbol{v_x^2 + v_y^2}}$$

**Viscosity estimation of protein droplets**

Combination of normal light and fluorescence illumination microscopy was performed using the inverted microscope equipped with a Digital Single-Lens Reflex (DSLR) camera (EOS 550D, Canon, Japan), and 20× dry objective (LD A-Plan 20×/0.35 Ph1, Zeiss, Germany). The microparticles were added to 20 µL of the protein solution and then applied on a glass slide (VWR, USA). Prior to any

interaction with the particle-protein mixture, the electromagnetic needle was demagnetized by applying sinusoidal decaying signal to the coil (5), where $A = 0.25$ (V) is the amplitude, $F = 20$ (Hz) is sinusoidal frequency, $\varphi = 0$ is the phase shift, $a = 4$ is the attenuation factor, and $t = 3.33$ (µs) is the sampling period of analog-to-digital/digital-to-analog (AD/DA) converter (NI 6343, National Instruments, USA) from which the signal was produced. After the demagnetization, the needle position was calibrated by bringing it into contact with the glass slide and then moving it up (vertical z direction) for 100 µm. The protein-particle mixture was then applied and the scene was navigated in order to find particles encapsulated within the protein droplets. Once the particle(s)-in-protein coacervates were identified, the needle was brought into the vicinity and the excitation current of 350mA was applied. Each excitation signal lasted for 3 seconds. The particles in the coacervate droplets started to move towards the electromagnetic needle after experiencing the magnetic field gradient originating from the needle. The movements of particles were recorded with the DSLR camera. In similar fashion as before, the trajectories of the moving particles were obtained by a tracking algorithm based on the Hough Transform and implemented in MATLAB. The position data along each axis was smoothed with smoothing spline ($R^2 \geq 0.99$) followed by derivation, i.e. obtaining $v_x$ and $v_y$. The magnitude of the velocity vector was computed according to equation (4) and the protein droplet viscosity was estimated by using the equation (6).

(5)

$$\boldsymbol{y = A \sin(2\pi F t + \varphi) e^{-at}}$$

(6)

$$\boldsymbol{\eta_{pd} = \frac{F_m}{3\pi d v}}$$

**Inverse capillary velocity determination and surface tension estimation.**

The procedure for determining the inverse capillary velocity was adopted from Brangwynne *et al.* 2011[31]. The aspect ratio (A.R.) of the protein droplets was determined by fitting an ellipse to the droplet contour and calculating the aspect ratio according to equation (7) where $l_{long}$ and $l_{short}$ are the major long and short axes of the ellipse. For analysis of the merging droplets, the time evolution of this aspect ratio was fitted to function (8) , where t is time, τ is the characteristic relaxation time, and $A.R._0$ is the initial aspect ratio. We calculated the radius $l$ of the final uniform droplet. Plots of τ vs. ℓ were fitted to a line ($a \cdot x$), to determine the inverse capillary velocity (9).

(7)

$$\boldsymbol{A.R.} = \boldsymbol{l_{long}} / \boldsymbol{l_{short}}$$

(8)

$$\boldsymbol{A.R.} = \boldsymbol{1} + (\boldsymbol{A.R._0} - \boldsymbol{1}) \cdot \boldsymbol{e}^{-\frac{t}{\tau}}$$

(9)

$$\frac{\boldsymbol{\tau}}{\boldsymbol{l}} = \frac{\boldsymbol{\eta}}{\boldsymbol{\gamma}}$$

**LLC films**

10 µL of LLC solutions (either 2% w/v or 30% w/v) were spread over Parafilm to an area of 5mm × 5mm. LLC solutions were exposed to air for 2 min to allow evaporation of water to form a semi-dried LLC film on the surface of Parafilm before stretching it at the rate of approximately 1 mm $s^{-1}$ up to 200%.

**Single filament pulling from LLCs**

Series of stepwise concentrations were carried out to prepare LLC for filament pulling. In the first step the total concentration of dilute protein solution was gradually increased to approximately 2% w/v using Vivaspin centrifugal concentrators (Sigma-Aldrich) at 25̊C and 840-1500 r.c.f (Gradual concentrating). During concentration, the dense LLC phase was collected. In the second step (Semi-gradual concentrating), samples were further concentrated to approximately 30% w/v (storage concentration for the LLC solution) using SpeedVac concentrator. Due to the long processing time, samples were flash-frozen in between with liquid nitrogen. In the third step (Cleaning step), non-specific aggregates, fibrils, and gel-particles were removed from the solution by centrifugation and

careful removal of large visible pieces. Additionally samples where filtered through PVDF membranes with 0.22 µm pore size (Millipore Millex-GV).

For pulling a filament, 10-15 µl of the 30% w/v LLC solution was placed between the narrow tips of tweezers and rapidly concentrated further to 70-75% w/v by evaporation in air flow. Filament formation was carried out in following three steps. In the first step immature fiber was formed by constant pulling and stretching of highly concentrated LLC solution under airflow at the rate of roughly 1 mm $s^{-1}$ to form a 1 cm in length filament. This was considered as 0% post-stretched filament (or immature filament). In the second step, more water was allowed to evaporate for approximately 30-40 s. In the third step filaments were post-stretched either 100% or 150% of their initial lengths at half of the initial pulling rate (0.5 mm $s^{-1}$). Filaments were then kept under tension to dry completely for minimum of 2 hours at ambient conditions to form mature filaments for tensile testing and wide angle X-ray scattering measurements.

**Molecular dynamic (MD) simulation**

Four antiparallel α-helices were generated with CHIMERA. TLEAP in ANTECHAMBER was used to create force field parameters for the protein (ff14SB), add hydrogens, and solvate the ligand-protein complex with a rectangular box of transferable intermolecular potential three-point water molecules (TIP3P) 10 Å in all directions. The MD simulation was run with SANDER module within AMBER 14 with the following protocol. The system was first minimized with conjugate-gradient method for 1000 steps without restraints. This was followed by an equilibration step at constant volume by allowing the system to heat from 100 to 300 K for 1000 steps with NMR restraints. The production simulation without restraints was run for 128,000 steps (simulation time 256 ps) at constant pressure controlled by isotropic position scaling. Temperature was maintained with the Berendsen thermostat, applied with a heat bath coupling time of 0.2 ps. Electrostatics were treated with Particle-Mesh Ewald (PME) method and cutoff value of 12 Å for non-bonded interactions was employed. The equilibration step and the production simulation were run under periodic boundary conditions. The SHAKE algorithm was used to restrain bonds involving hydrogen atoms, allowing the use of 2 fs time step.

**Steered molecular dynamic simulation (SMD)**

NAMD was used for the SMD. $C^{\alpha}$ atoms of GLY 1 of chains A and C, in addition to $C^{\alpha}$ atoms of GLY13 of chains B and D were kept fixed. An external force was applied to the center of mass of the $C^{\alpha}$ atoms of GLY13 of chains A and C, and GLY1 of chains B and D. The direction of the constant velocity (2.5Å/ps)

was defined by the vector of $C^{\alpha}$ atoms of GLY1 and GLY13 of chain A. The 20,000 step simulation was performed at 300 K in vacuum. The SHAKE algorithm was used to restrain bonds involving hydrogen atoms and the time step used in SMD production simulations was 2 fs.

**Simulated WAXS scattering**

The theoretical WAXS scattering curves for the simulated structures were calculated using the program CRYSOL. The coordinates derived from the MD simulation trajectories were used as input for the software. WAXS curves in the form of log(I/Io) versus Q were generated by CRYSOL using a hydration shell of 0.334 e/$Å^{-3}$.

**Pyriform inspired adhesive attachment discs**

To mimic the natural pyriform discs of spiders, a wet-spun pure native form cellulose nanofibril fiber (CNF) was attached on acrylic glass substrate by approximate seventy spun filaments of CBM-ADF3-CBM distributed and packed over 2 mm length (Fig. 3C and Fig. S2). To mount the samples, the end of the CNF fiber on the mobile side was fixed with fast-cold-curing Loctite® adhesive on acrylic glass and the other end of the CNF fiber attached on the substrate with the spun filaments to the fixed part of the sample holder. To measure the forces of the adhesive attachment , the same micro-mechanical tensile tester was used. The pulling rate was set to 10 µm/second and the force versus extension data was recorded.

**Supplementary References**


1. Baldwin, R. L. How Hofmeister ion interactions affect protein stability. *Biophys. J.* **71,** 2056–2063 (1996).
2. Record, M. T., Guinn, E., Pegram, L. & Capp, M. Introductory lecture: interpreting and predicting Hofmeister salt ion and solute effects on biopolymer and model processes using the solute partitioning model. *Faraday Discuss.* **160,** 9–44 (2013).
3. Simmons, A. H., Michal, C. A. & Jelinski, L. W. Molecular Orientation and Two-Component Nature of the Crystalline Fraction of Spider Dragline Silk. *Science* **271,** 84–87 (1996).
4. Lefèvre, T., Rousseau, M.-E. & Pézolet, M. Protein secondary structure and orientation in silk as revealed by Raman spectromicroscopy. *Biophys. J.* **92,** 2885–2895 (2007).
5. Kümmerlen, J., Van Beek, J. D., Vollrath, F. & Meier, B. H. Local structure in spider dragline silk investigated by two-dimensional spin-diffusion nuclear magnetic resonance. *Macromolecules* **29,** 2920–2928 (1996).
6. Vollrath, F. & Knight, D. P. Liquid crystalline spinning of spider silk. *Nature* **410,** 541–8 (2001).
7. Tremblay, M.-L. *et al.* Spider wrapping silk fibre architecture arising from its modular soluble protein precursor. *Sci. Rep.* **5,** 11502 (2015).
8. Deravi, L. F., Golecki, H. M. & Parker, K. K. Protein-based textiles: bio-inspired and bio-derived materials for medical and non-medical applications. *J. Chem. Biol. Interfaces* **1,** 25–34 (2013).
9. Su, I. & Buehler, M. J. Nanomechanics of silk: the fundamentals of a strong, tough and versatile material. *Nanotechnology* **27,** 302001 (2016).
10. Giesa, T., Perry, C. C. & Buehler, M. J. Secondary structure transition and critical stress for a model of spider silk assembly. *Biomacromolecules* **17,** 427–436 (2016).
11. Landreh, M., Johansson, J., Rising, A., Presto, J. & Jörnvall, H. Control of amyloid assembly by autoregulation. *Biochem. J.* **447,** 185–192 (2012).
12. Johansson, J., Nerelius, C., Willander, H. & Presto, J. Conformational preferences of non-polar amino acid residues: an additional factor in amyloid formation. *Biochem. Biophys. Res. Commun.* **402,** 515–518 (2010).
13. van Beek, J. D., Hess, S., Vollrath, F. & Meier, B. H. The molecular structure of spider dragline silk: folding and orientation of the protein backbone. *Proc Natl Acad Sci U S A* **99,** 10266–10271 (2002).
14. Henzler Wildman, K. A., Lee, D. & Ramamoorthy, A. Determination of α-helix and β-sheet stability in the solid state: A solid-state NMR investigation of poly (L-alanine). *Biopolymers* **64,** 246–254 (2002).
15. Rising, A. & Johansson, J. Toward spinning artificial spider silk. *Nat. Chem. Biol.* **11,** 309–315 (2015).

16. Rafik, M. E., Doucet, J. & Briki, F. The intermediate filament architecture as determined by X-ray diffraction modeling of hard α-keratin. *Biophys. J.* **86,** 3893–3904 (2004).

17. Loke, J. J., Kumar, A., Hoon, S., Verma, C. & Miserez, A. Hierarchical Assembly of Tough Bioelastomeric Egg Capsules is Mediated by a Bundling Protein. *Biomacromolecules* **18,** 931–942 (2017).

18. Partlow, B. P. *et al.* Silk Fibroin Degradation Related to Rheological and Mechanical Properties. *Macromol. Biosci. 2016,* **16,** 666−675 (2016).

19. Sampath, S. & Yarger, J. L. Structural hysteresis in dragline spider silks induced by supercontraction: an X-ray fiber micro-diffraction study. *RSC Adv.* **5,** 1462–1473 (2015).

20. Ulrich, S., Glisovic, A., Salditt, T. & Zippelius, A. Diffraction from the beta-sheet crystallites in spider silk. *Eur Phys J E Soft Matter* **27,** 229–242 (2008).

21. Sampath, S. *et al.* X-ray diffraction study of nanocrystalline and amorphous structure within major and minor ampullate dragline spider silks. *Soft Matter* **8,** 6713 (2012).

22. Miserez, A., Wasko, S. S., Carpenter, C. F. & Waite, J. H. Non-entropic and reversible long-range deformation of an encapsulating bioelastomer. *Nat. Mater.* **8,** 910–916 (2009).

23. Van Beek, J. D., Kümmerlen, J., Vollrath, F. & Meier, B. H. Supercontracted spider dragline silk: a solid-state NMR study of the local structure. *Int. J. Biol. Macromol.* **24,** 173–178 (1999).

24. Knowles, T. P. J., Vendruscolo, M. & Dobson, C. M. The amyloid state and its association with protein misfolding diseases. *Nat. Rev. Mol. cell Biol.* **15,** 384–396 (2014).

25. Jahn, T. R. & Radford, S. E. The Yin and Yang of protein folding. *FEBS J.* **272,** 5962–5970 (2005).

26. Huemmerich, D. *et al.* Primary Structure Elements of Spider Dragline Silks and Their Contribution to Protein Solubility. *Biochemistry* **43,** 13604–13612 (2004).

27. Guerette, P. a, Ginzinger, D. G., Weber, B. H. & Gosline, J. M. Silk properties determined by gland-specific expression of a spider fibroin gene family. *Science* **272,** 112–5 (1996).

28. Gosline, J. M., Guerette, P. a, Ortlepp, C. S. & Savage, K. N. The mechanical design of spider silks: from fibroin sequence to mechanical function. *J. Exp. Biol.* **202,** 3295–3303 (1999).

29. Kollmannsberger, P. & Fabry, B. High-force magnetic tweezers with force feedback for biological applications. *Rev. Sci. Instrum.* **78,** 1–6 (2007).

30. Lee, J. H. *et al.* Magnetic nanoparticles for ultrafast mechanical control of inner ear hair cells. *ACS Nano* **8,** 6590–6598 (2014).

31. Brangwynne, C. P., Mitchison, T. J. & Hyman, A. A. Active liquid-like behavior of nucleoli determines their size and shape in Xenopus laevis oocytes. *Proc. Natl. Acad. Sci.* **108,** 4334–4339 (2011).
