# Supplementary material for: Phase transitions as intermediate steps in the formation of molecularly engineered protein fibers
Source: Commun Biol. 2018 Jul 2;1:86. doi: 10.1038/s42003-018-0090-y (PMC6123624; doi:10.1038/s42003-018-0090-y)
Supplement: Supplementary file 2 — Description of Additional Supplementary Files [file 42003_2018_90_MOESM2_ESM.docx]

**Description of Additional Supplementary Files**

File Name: Supplementary Movie 1

Description: Inverted light microscopy of the dense phase showing how spherical liquid-like

coacervate (LLC) droplets coalesce, deform, and relax upon physical shearing.

File Name: Supplementary Movie 2

Description: Rapid formation of fibers by extending a 10-15 µl aliquot of LLC at 70-75% w/v concentration.

File Name: Supplementary Movie 3

Description: Self-amalgamation and adherence of two freshly pulled LLC fibersto each other.

File Name: Supplementary Movie 4

Description: Adhesive property of LLC fibers during in-plane adhesion force measurement in which a cellulosic based fiber anchored to a PMMA surface using multiple LLC fibers.

File Name: Supplementary Movie 5

Description: Normal light and fluorescence illumination microscopy of the magnetophoretic manipulation of an encapsulated red fluorescent superparamagnetic (Fe3O4@PS) particle in an LLC droplet of CBM-eADF3-CBM (the time when the current is ON or OFF is indicated in the video).

File Name: Supplementary Data 1

Description: Amino acid sequences of proteins used
